# Prostate Cancer Cell Extracellular Vesicles Increase Mineralisation of Bone Osteoblast Precursor Cells in an In Vitro Model

**DOI:** 10.3390/biology10040318

**Published:** 2021-04-10

**Authors:** Ben Lanning, Jason Webber, Pinar Uysal-Onganer, Wen Guo Jiang, Aled Clayton, Dafydd Alwyn Dart

**Affiliations:** 1 Cardiff China Medical Research Collaborative, Cardiff University School of Medicine, Cardiff CF14 4YS, UK; ben.lanning@hotmail.co.uk (B.L.); jiangw@cardiff.ac.uk (W.G.J.); 2 Tissue Microenvironment Group, Division of Cancer & Genetics, School of Medicine, Cardiff University, Cardiff CF14 4YS, UK; j.p.webber@swansea.ac.uk (J.W.); claytona@cardiff.ac.uk (A.C.); 3 Institute of Life Science, Swansea University Medical School, Swansea University, Singleton Park, Swansea SA2 8PP, UK; 4 School of Life Sciences, College of Liberal Arts and Sciences, University of Westminster, 115 New Cavendish Street, London W1W 6UW, UK; p.onganer@westminster.ac.uk; 5 Institute of Medical and Biomedical Education, St George’s University of London, Cranmer Terrace, Tooting, London SW17 0RE, UK

**Keywords:** prostate, cancer, bone, metastasis

## Abstract

**Simple Summary:**

Prostate cancer frequently metastasizes to the bone, where it forms primarily osteoblastic lesions. Currently there is no real therapeutic option for this late stage of disease, and understanding prostate cancer-bone interaction and communication is vital. Using a simple in vitro model of os-teoblast differentiation and mineralization, we studied this interaction and observed that prostate cancer cells secreted large quantities of extracellular vesicles containing microRNAs. When ex-posed to the extracellular vesicles, increased osteoblast differentiation and mineralization could be observed, and upon RNA-seq several of these microRNAs were implicated as upstream regulators of the mineralization process. These microRNAs also correlated with poor survival in online analysis of patient datasets. We characterized and validated four genes known to be targeted by microRNA-16, and found that extracellular vesicles could deliver miR-16, and increase minerali-zation.

**Abstract:**

Skeletal metastases are the most common form of secondary tumour associated with prostate cancer (PCa). The aberrant function of bone cells neighbouring these tumours leads to the devel-opment of osteoblastic lesions. Communication between PCa cells and bone cells in bone envi-ronments governs both the formation/development of the associated lesion, and growth of the secondary tumour. Using osteoblasts as a model system, we observed that PCa cells and their conditioned medium could stimulate and increase mineralisation and osteoblasts’ differentiation. Secreted factors within PCa-conditioned medium responsible for osteoblastic changes included small extracellular vesicles (sEVs), which were sufficient to drive osteoblastogenesis. Using MiR-seq, we profiled the miRNA content of PCa sEVs, showing that miR-16-5p was highly ex-pressed. MiR-16 was subsequently higher in EV-treated 7F2 cells and a miR-16 mimic could also stimulate mineralisation. Next, using RNA-seq of extracellular vesicle (EV)-treated 7F2 cells, we observed a large degree of gene downregulation and an increased mineralisation. Ingenuity® Pathway Analysis (IPA^®^) revealed that miR-16-5p (and other miRs) was a likely upstream effec-tor. MiR-16-5p targets in 7F2 cells, possibly involved in osteoblastogenesis, were included for val-idation, namely AXIN2, PLSCR4, ADRB2 and DLL1. We then confirmed the targeting and dow-regulation of these genes by sEV miR-16-5p using luciferase UTR (untranslated region) reporters. Conversely, the overexpression of PLSCR4, ADRB2 and DLL1 lead to decreased osteoblastogene-sis. These results indicate that miR-16 is an inducer of osteoblastogenesis and is transmitted through prostate cancer-derived sEVs. The mechanism is a likely contributor towards the for-mation of osteoblastic lesions in metastatic PCa.

## 1. Introduction

Prostate cancer (PCa), the most common form of cancer to occur in men [1], can metastasise to distal sites, with the bone being the commonest metastatic location (84%) [2]. PCa cells frequently infiltrate the trabecular bones as well as proximal ends of the femurs—but the reasons for the preference is unknown.

The bone matrix consists of hydroxyapatite (Ca_5_(PO_4_)_3_(OH)), and organic material including cells, collagenous proteins and growth factors [3]. Three principal cell types exist in the bone including osteoblasts, osteoclasts and osteocytes. The main role of the osteoblasts is the creation of fresh bone matrix, by producing the organic matrix (osteoid) and then the mature mineralised bone matrix. Osteoblastogenesis from mesenchymal stem cells is controlled by Wnt and bone morphogenic protein (BMP). The genes *RUNX2*, *DLX5* and *OSTERIX* then elicit functional protein expression, e.g., alkaline phosphatase and collagen type 1.

Bone remodelling involves bone activation, resorption, reversal, and matrix deposition. PCa produces mainly osteoblastic tumours (85%), with remaining mixed lesions and osteolytic lesions [4,5]. To define PCa metastasis as mainly osteoblastic is an oversimplification, as general remodelling with regions of osteolytic and osteoblastic growth occur simultaneously. Osteolytic effects may be required ahead of osteoblastic growth as cells require access to the bone. Osteoblastic bone lesions are characterised by the overactivity of osteoblasts resulting in excess bone deposition, detectable on X-ray imaging. PCa cells secrete factors such as BMP, TGF-β (Transforming growth factor beta), and IGF (Insulin-like growth factor), which trigger osteoblastogenesis, by activating genes, e.g., *RUNX2*, *OSTERIX*. However, it is clear that multiple factors including other growth factors, hormones, nucleic acids, lipids, and adhesion molecules contribute in a coordinated fashion to direct bone remodelling. How PCa cells orchestrate such complexity is not completely understood.

Recent evidence suggests that cancer cells can signal via other pathways, including through the secretion of extracellular vesicles (EVs) that carry and deliver growth factors, microRNAs and other factors to recipient cells [6,7].

Small RNA, in particular microRNAs, are relatively abundant within EVs and EV-mediated nucleic acid cell-to-cell transfer has been described [8,9]. EVs’ constituents are derived from their cell of origin, including proteins and nucleic acids (mRNA and miRNAs) [2,3,4]. Whilst there is mounting evidence detailing that the mRNA content of EVs can alter significantly in cancer, and hence provide some utility as disease markers, there is also growing interest in the functionality of such molecules, acting as either signalling factors in their own right or as modulators of the cells’ transcriptome.

Prostate cancer cell-derived extracellular vesicles and their cargo of mRNA and miRNA are becoming important signalling molecules which have important roles in metastasis formation. EV-derived miR-141-3p has been shown to regulate osteoblast activity [10] and that EV-derived miR-940 can induce osteoblastic phenotypes in the bone metastatic microenvironment [11]. EV-derived mRNA and miRs has been implicated in the potential mechanism of metastasis to the bone [12].

Herein, we have investigated an in vitro osteoblastogenesis model system to explore the possible role of prostate cancer cell-derived EVs in bone lesions. Here, we identify an abundant EV-encapsulated miRNA (miR-16-5p) that stimulates bone mineralisation formation and recognise EV-transmitted miR-16-5p as an important component to the dysregulation of the bone environment, with implications for osteoblastic prostate cancer metastasis.

## 2. Materials and Methods

### 2.1. Cell Culture

LNCaP cells were maintained in RPMI medium (Sigma, Gillingham, Dorset, UK). RAW-264, PC3, VCaP and Du145 (and variants Du145 ^Rab27ko^ [13]) cells were maintained in DMEM medium (Sigma). Media was supplemented with 2 mM L-glutamine, 100 units/mL penicillin, 100 mg/mL streptomycin (Sigma) and 10% foetal bovine serum (First Link, UK). RWPE-1 (HPV-18 immortalized normal adult human prostate), PZ-HPV7 (HPV-18 immortalised prostate epithelial cells), CA-HPV-10 (immortalized human prostatic epithelial cells), PNT1A (SV40 immortalised normal human prostate cells) and PNT2C2 (SV40 immortalised normal human prostate cells) were grown in keratinocyte serum free medium (Gibco) supplemented with bovine pituitary extract (0.05 mg/mL) and human recombinant EGF (Epidermal Growth Factor) (5 ng/mL) (Gibco). 7F2 bone marrow osteoblast precursor cells were grown in αMEM (Minimum Essential Media) with 2 mM L-glutamine, 1 mM sodium pyruvate and 10% foetal bovine serum. Cells were obtained from the ATCC (American Type Culture Collection) in 2017 and were used within 10 passages.

For spheroids, cells were grown in 96 well round bottomed plates (Nunc).

### 2.2. Co-Culture Assays

7F2 cells were plated in 24 well plates. Prostate cancer cells were cultured in an upper transwell chamber (Corning) with a 0.4 µm permeable membrane. Media was 50:50 DMEM: αMEM.

### 2.3. Osteoblast Mineralisation

7F2 cells treated with osteogenic media (MEM + 5 mM β-glycerophosphate, 0.1 g/L ascorbic acid, 10^−8^ mol/L menadione and 10^−7^ mol/L 1.25(OH)_2_D3), as described in Guo et al., 2014 [14].

### 2.4. sEV Isolation and Nano Particle Analysis

EVs were purified from the supernatant by the 30% sucrose/D_2_O cushion method as described in Mitchell et al., 2008 [15]. Nanoparticles were quantified, and size distribution profiles determined by nanoparticle tracking analysis (NanoSight NS300 system). The full method is described in the Supplementary methods section.

### 2.5. Cellular Staining Assays

#### 2.5.1. Alizarin Staining for Calcium

Cells were washed with PBS (phosphate buffered saline), fixed in 10% formalin (30 min) and then washed again. Cells were incubated in Alizarin Red (2% *w*/*v*, pH 4.2) for 45 min, and washed 4 times with water. For quantification, Alizarin Red stain was eluted in 10% acetic acid, and the absorbance (450 nm) measured.

#### 2.5.2. Crystal Violet Assay for Cell Number

Cells were fixed in 10% formalin (30 min), washed and stained with crystal violet (0.5% *w*/*v*) for 30 min, and washed again. Cells were visualised, or the crystal violet was then dissolved in 10% acetic acid and absorbance (600 nm) using a GloMax plate reader (Promega, Madison, WI, USA).

#### 2.5.3. Alkaline Phosphatase (AP) Stain

Cells were fixed in 10% formalin for 1min and washed. BCIP/NBT (5-bromo,4-chloro,3-indolylphosphate/Nitro-Blue Tetrazolium) solution was added and incubated in the dark for 10 min, and then washed.

#### 2.5.4. Von Kossa Stain for Phosphate (or Calcium Phosphate)

Cells were fixed in 10% formalin for 30 min and then washed in water. A concentration of 1% (*w*/*v*) silver nitrate was added and incubated for 1 h under UV light, and then washed with water. Excess silver nitrate was removed with Na_2_S_2_O_3_ (5% *w*/*v*), and then washed in water.

### 2.6. Western Blotting

Protein lysates were prepared from cell samples using RIPA lysis buffer (radio-immunoprecipitation assay buffer), and measured using the BioRad protein quantification assay.

### 2.7. Transfection and Luciferase Assays

Cells were transfected with firefly luciferase UTR reporters, and constitutive expression renilla vectors (pGL4.75, Promega), using Lipofectamine 3000 (Life Technologies). Once 24 h had passed after transfection, cell lysates were mixed with *D*-luciferin substrate (Promega), and then with renilla substrate (Promega). Light emission was measured via a Glomax luminometer (Promega). Luciferase activity was normalised to renilla.

### 2.8. RNA Extraction and RT-PCR

Total RNA samples were prepared using Trizol reagent (Sigma) and converted to cDNA using the GoScript^™^ Reverse Transcription System (Promega). The advanced miRNA reverse transcription kit (ThermoFisher, Waltham, MA, USA) was used for all miR assays. For RNA extraction from liquid media, Trizol-LS was used. 

### 2.9. Q-PCR

Reactions were performed in triplicate on cDNA samples in 96-well optical plates on an ABI Prism StepOne System (ThermoFisher, U.K.). It consisted of 2 μL of cDNA, 7 μL of PCR-grade water, 10 μL of 2 × TaqMan Universal PCR Master Mix (Applied Biosystems, Foster City, CA, USA), 1 μL of Taqman specific assay probes (Applied Biosystems). Data were recorded using SDS software (v 2.3; PE Applied Biosystems). Advanced miRNA Taqman probes (ThermoFisher) were used for all miR qPCR assays.

### 2.10. RNA-seq Analysis

PolyA mRNA was isolated from total RNA using the Dynabeads mRNA DIRECT Kit (Life Technologies, UK) and verified using a Bioanalyser-2100 (Agilent, Santa Clara, CA, USA). RNAseq was carried out essentially as described in Dart et al., 2017 [16]. For MiR-seq we utilised NEXTFlex Small RNA-seq kit (Bioo Scientific, Austin, TX, USA).

## 3. Results

### 3.1. Characterisation of Mineralising Osteoblasts

There are several stages associated with bone metastasis-invasion, local debulking, tumour growth and bone cell growth stimulation [4,5], creating microenvironments that increase osteoblastogenesis.

**Figure 1:**
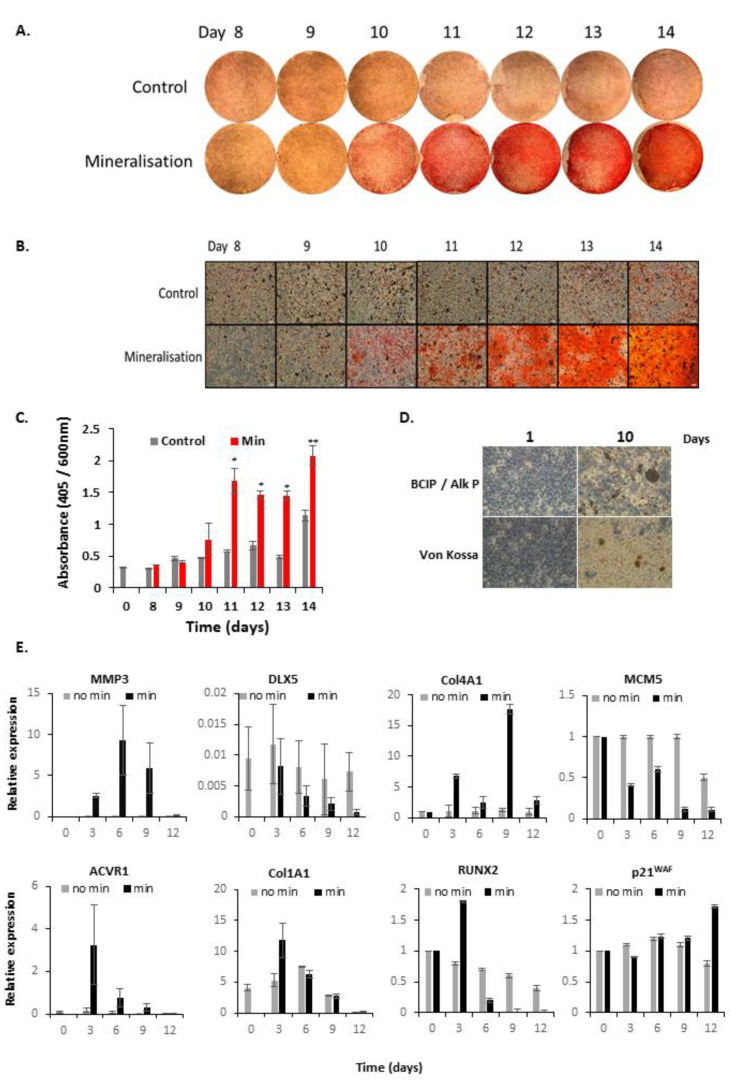
**Characterisation of Mineralising 7F2 Osteoblasts.** 7F2 bone marrow osteoblasts were stimulated to mineralise in osteogenic media containing 5 mM β-glycerophosphate, 0.1 g/L ascorbic acid, 10^−8^ mol/L menadione and 10^−7^ mol/L vitamin D3. (**A**), Whole well images of 7F2 cells grown in regular (control) media or osteogenic media containing over 14 days stained with alizarin red. (**B**), microscopy images of alizarin red stained 7F2 cells. (**C**), Quantification of alizarin red stained 7F2 cells treated with or without osteogenic medium for 14 days. Graph shows alizarin red absorbance (405 nm) normalised to a post stain crystal violet stain (600 nm) for cell number. (**D**), Representative image of alkaline phosphatase staining and Von Kossa staining of 7F2 cells treated with osteogenic media for 1–10 days. (**E**), QPCR analysis for gene expression of a panel of genes involved in osteoblastogenesis or the cell cycle, from 7F2 cells treated with or without osteogenic media for 12 days. Data is normalised to housekeeping genes β−actin, GAPDH and RPL19. * *p* = <0.05, ** *p* = <0.01.

Osteoblasts produce bone via the production of organic bone matrix proteins (collagens) and then by their mineralisation. To model this, we utilised 7F2 cells—spontaneously immortalized cells from p53^−/−^ mouse bone marrow. In osteogenic medium they mineralised their matrix over 10 days approx. [17,18]—assessed by alizarin red staining for Ca^2+^, von Kossa staining for PO_4_^3-^ and BCIP staining for alkaline phosphatase activity. Figure 1A,B, shows the alizarin red staining, seen over 14 days. Mineralisation, quantified by the absorbance of dissolved alizarin red stain, became statistically significant at day 11. Cell numbers were normalised by crystal violet assay, as shown in Figure 1C. Alkaline phosphatase and von Kossa staining demonstrated increased mineralisation at this time (see Figure 1D).

Mineralisation represents the phenotypic end-stage, but we were also interested in earlier genetic changes in response to hormone treatments. Cells were collected over 10 days and analysed by Q-PCR for genes involved in osteoblast mineralisation, including *MMP3, DLX5, Col4A1, ACVR1, COL1A1, RUNX2* (see Figure 1E). *p21^WAF^*(*CDKN1A*) and *MCM5* were studied as markers of cell cycle exit during differentiation.

MMP3 (matrix metalloproteinase-3) is an extracellular matrix remodelling enzyme, which peaked at day 6. A regulator of osteoblastogenesis, the gene DLX5 (distal-less homeobox 5) decreased in expression. Collagens (COL1A1 and Col4A1) are expressed in osteoblasts during early bone matrix formation. *COL1A1* showed a statistically significant increase at day 3, and *Col4A1* on day 9. *ACVR1*, a receptor of bone morphogenic protein (BMP7), peaked at day 3. The cell cycle inhibitor *p21^waf1^* (*CDKN1A*) increased over time, whilst *MCM5* strongly decreased. Overall, these changes are consistent with the onset of bone remodelling and differentiation with cells exiting the cell cycle.

### 3.2. PCa Cells Stimulate 7F2 Osteoblast Mineralisation

In mouse models, PC3 and VCaP cells frequently metastasise to the bone, whereas LNCaP cells do so rarely, or as aggressive subclones. PC3 cells have been shown to cause primarily osteolytic tumours in mice, contrary to most prostate cancer bone metastasis, however, PC3 subclones cells have been shown to produce mixed lesions with osteolytic and osteoblastic tumour induction within the same site [19]. Many PCa cells, including PC3, can stimulate osteoblast cell growth and differentiation [12,20], and can interact and modify bone growth. To test how these cell lines affect the osteoblastic process, we utilised the 7F2 mineralisation model, using various co-culture experiments.

**Figure 2:**
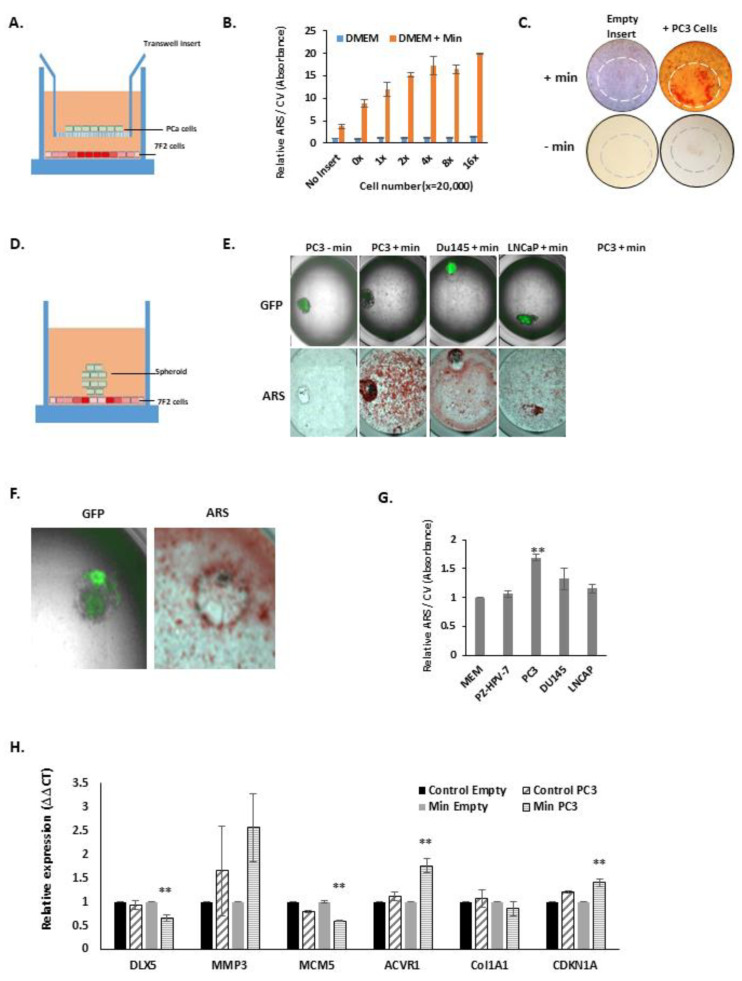
**Prostate Cancer cells stimulate 7F2 Osteoblastic Mineralisation**. (**A**), A schematic diagram of the cell co-culture system, including the transwell insert. (**B**), Alizarin red staining of 7F2 cells co-cultured with increasing numbers of PC3 cells either with or without osteogenic medium seeded in the co-culture insert. Graph shows alizarin red absorbance (405 nm) normalised to a post stain crystal violet (CV) stain (600 nm) for cell number. Graph shows average fold change from three independent experiments compared to 0 PC3 cells and no osteogenic media. (**C**), Images of the whole well of 7F2 cells stained with alizarin red after incubation with PC3 transwell insert with or without osteogenic media. Circle inset grey (hatched line) shows the outline of the insert in relation to the holding well. (**D**), A schematic of the spheroid-7F2 model. (**E**), Images of 7F2 cells incubated with osteogenic media and with a GFP-expressing prostate cancer cell line spheroids placed upon the monolayer. Cells were first imaged using the GFP wavelength (470/525nm) upper panel, before spheroid was removed and 7F2 cells were stained with alizarin red, lower panel. (**F**), magnified image of PC3 GFP expressing spheroid placed on mineralisation 7F2 cells. Note the ring of mineralisation. (**G**), Effect of conditioned media from various prostate cancer cells lines on 7F2 mineralisation. Cells were mineralised for 10 days in conditioned media supplemented with osteogenic components. Mineralisation was normalised to that obtained with osteogenic MEM. Graph shows alizarin red absorbance (405nm) normalised to a post stain crystal violet stain (600nm) for cell number. Average from three independent experiments is shown + SEM. (**H**), Q-PCR analysis of osteoblast differentiation gene expression (or cell cycle genes) from 7F2 cells co-cultured with a transwell chamber containing PC3 cells (or empty chamber = empty) with (min = osteogenic mineralisation media) or without osteogenic medium (control). Data is normalised to housekeeping genes β-actin, GAPDH and RPL19. ** *p* = <0.01.

Increased 7F2 mineralisation was seen when co-cultured with PC3 cells in a transwell chamber for up to 10 days (see Figure 2A,B), increasing with PC3 cell number (see Figure 2B). An increase in overall mineralisation was seen, but also a cluster of increased mineralisation was seen in those cells immediately under the insert—as visualised by alizarin red staining (see Figure 2C). A detectable amount of mineralisation was seen in the 7F2 cells directly underneath the transwell insert without osteogenic media (Figure 2C lower panel).

We then studied how PCa cells directly interacted and modulated 7F2 cell mineralisation. GFP^+ve^ PCa cell spheroids were grown and transferred to mineralising 7F2 cells and incubated for 5 days (see Figure 2D). When PC3, Du145 and LNCaP spheroids physically interacted with mineralising 7F2 cells, an area of increased mineralisation was observed in the immediate spheroid vicinity (see Figure 2E). In the absence of mineralising hormones, the PC3 spheroid had disrupted the 7F2 monolayer, and did not cause mineralisation. Interestingly, in some instances the PC3 cells produced a thin ring of mineralisation on the outside edge of the spheroid, but reduced under the main spheroid body (see Figure 2F).

We then studied whether PCa mineralisation effects were due to secreted signalling molecules, by collecting PCa conditioned media and using it to treat 7F2 cells along with mineralising hormones (see Figure 2G). PC3 and Du145 conditioned media stimulated the mineralisation of 7F2 cells, with PC3 being the strongest and most significant. LNCaP conditioned medium showed a modest stimulation, whereas conditioned medium from immortalised normal prostate epithelial cells, e.g., PZ-HPV-7, grown at the same density, failed to influence mineralisation.

Further, we analysed the effect of co-culture of PC3 on the expression of the mineralisation-related genes *MMP3, DLX5, COL1A1* and *ACRV1* in 7F2 cells, after 3 days (see Figure 2H). With PC3 coculture the expression of *DLX5* decreased, whilst the expression of *MMP3*, *ACVR1* increased. *Col1A1* remained relatively unchanged. The cell cycle inhibitor p21^waf1^ (*CDKN1A*) showed an increase and the DNA replication gene *MCM5* showed a strong decrease, indicative of differentiation.

### 3.3. PCa Cells Secrete miRNAs Both in Extracellular Vesicles and as Free Oligos

PCa cells influenced the 7F2 mineralisation when in direct physical contact, or via secreted molecules present in conditioned medium (that can transit transwell membranes). Proteins, e.g., growth factors, have previously been implicated in the mineralisation of osteoblastic metastasis, but we were interested in identifying the role of microRNAs.

**Figure 3:**
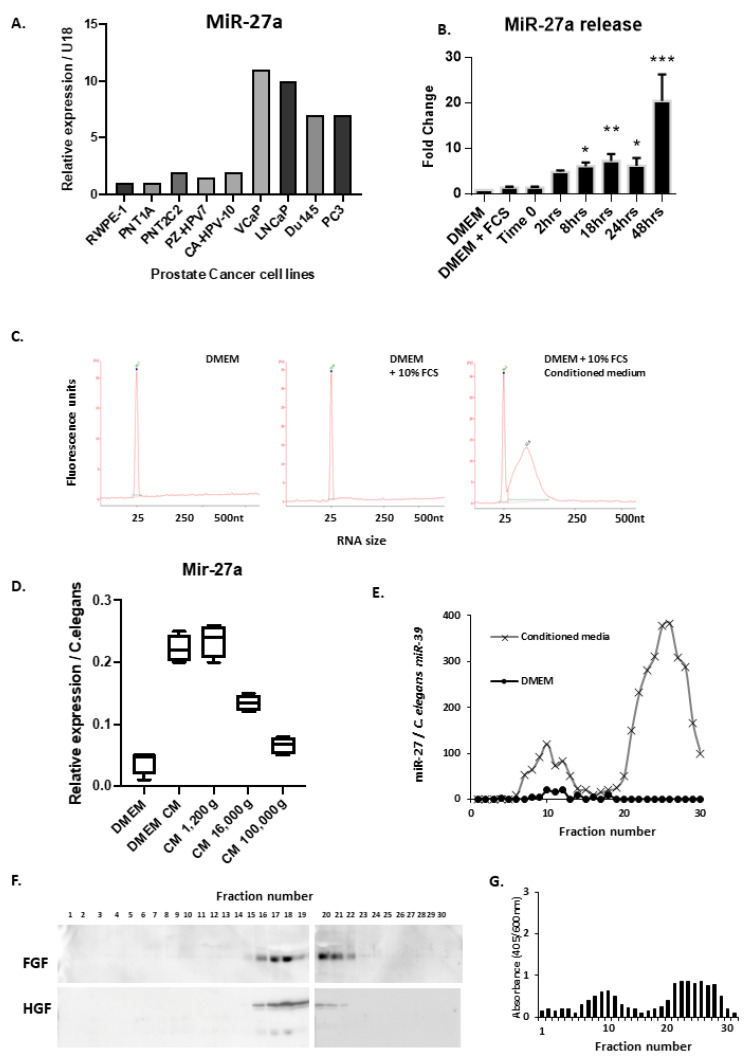
Prostate Cancer cells secrete large quantities of miRNAs both as Extracellular vesicles and as free or protein-bound miRs. (**A**), Q-PCR analysis of miRNA-27a expression from a panel of prostate cancer or normal prostate derived immortalised cell lines. Data is normalised to U18 small nuclear RNA and plotted relative to the RWPE-1 cell line. (**B**), Q-PCR analysis of miR-27a release into conditioned media form PC3 cells over 48 h. Data is normalised to U18 small nuclear RNA. (**C**), RNA extraction and bioanalysis of RNA from media (DMEM), media with FCS and conditioned media from PC3 cells after 48 h incubation. (**D**), Q-PCR analysis of miR-27a from RNA extracted from media and conditioned media after being subjected to increasing forces of centrifugation. (**E**), Q-PCR analysis of RNA extracted from DMEM or conditioned media from PC3 cells subjected to size exclusion chromatography, over 30 × 500μL fractions. RNA was extracted from each fraction. Data was normalised to a *C. elegans* miR-39 spike-in oligo, given at the time of RNA extraction. (**F**), Western blot analysis of each fraction from the size exclusion chromatography of PC3 conditioned medium. (**G**), Alizarin red assay for osteoblast mineralization of 7F2 cells using conditioned medium from PC3 cells. Graph shows alizarin red absorbance (405nm) normalised to a post stain crystal violet stain (600 nm) for cell number. * *p* = <0.05, ** *p* = <0.01, *** *p* = <0.001.

Firstly, we examined the PCa cells’ micoRNA secretion into conditioned media by plating equal numbers of cells into fresh media. Media was collected at intervals and RNA was extracted. We analysed the levels of the microRNA, miR-27a, in the conditioned media, as we had previously found this miR to be highly expressed in prostate cancer cell lines [21]. MiR-27a secretion was greatest from VCaP, LNCaP, Du145 and PC3 cells. The immortalised human prostate cells-RWPE1, PNT1A, PNT2C2, PZ-HPV-7 and CA-HPV-10 cells showed lower levels of secreted miR-27a (see Figure 3A). Similar results were seen for miRs-132-see Supplementary Figure S1.

Over 48 h, miR-27a secretion increased into conditioned media (20-fold) (see Figure 3B). Similar results were observed with Du145 cells (data not shown). Using a synthetic miR-27a oligo, a standard curve of miR_[conc.]_ vs. Ct_[val]_ was generated to calculate the concentration of secreted miRs. MiR-27a reached 5–50 pM after 48 h from approx. 100,000 PC3 cells (see Supplementary Figure S2). Bioanalysis showed the conditioned medium contained large quantities of <150 nt RNA (see Figure 3C).

To analyse whether miRs were produced in extracellular vesicles or as free oligos, we ultracentrifuged the conditioned media to pellet out any vesicles. RNA was then extracted from the supernatant. Ultracentrifugation removed 70% (approx.) of the miR-27a, indicating a significant portion was indeed vesicularly associated (see Figure 3D).

Secondly, size exclusion chromatography was carried out on the conditioned media in order to fractionate the components and separate vesicles from soluble components. Each fraction was subjected to RT-qPCR. Larger vesicles were eluted earlier in the fraction series, whereas smaller soluble molecules were eluted in later fractions. Chromatography revealed that miRs were present in two populations; associated with large molecular weight bodies (fractions 7–12) and as small molecular weight entities (fractions 18–25), as shown in Figure 3E. No significant miRs were detectable in DMEM. Conditioned media from Du145 cells showed a similar pattern, however Du145^Rab27ko^ cells (which have attenuated vesicles secretion, Webber et al., 2015) showed an absence at the first peak (fractions 7–12/vesicular) and a reduced miR-27a (and miR-21) level in the conditioned media, (see Supplementary Figure S1). This would be consistent with a dependency for secretion of miR-27a on endosome-derived vesicles (i.e., exosomes) as opposed to plasma membrane-derived microvesicles.

The chromatography column was calibrated by spiking in a synthetic miR (*C. elegans* miR-39) oligo, or purified sEVs (containing miR-27a), and the fractions analysed. The sEVs spike-in increased miR purification with the first peak (fractions 7–12) and the free oligo increased miR levels at the second peak (fractions 18–25). This showed clearly that two populations of miR existed, sEV-associated and sEV non-associated (see Supplementary Figure S1). Protein quantification from each fraction clearly showed that the soluble proteins eluted late from the column (fractions 20–28), see Supplementary Figure S1. Western blotting of each fraction showed FGF (Fibroblast growth factor) and HGF (Hepatocyte growth factor) eluted late from the column, and were separate from the sEV carrying fractions, as shown in Figure 3F.

Treating 7F2 cells with each fraction (and osteogenic hormones), showed that both sets of fractions 7–12 and 19–26 could increase the mineralisation of these cells (see Figure 3G).

In conclusion, the secretome of prostate cancer cells includes a vesicularly associated miR component, that is related to endosomal transport, and is physically distinct from a more soluble form of miR. Both vesicle and avesicular compartments, however, retain the promineralisation function when added to 7F2 cells.

### 3.4. sEV Characterisation and miR Cargo Identification

**Figure 4:**
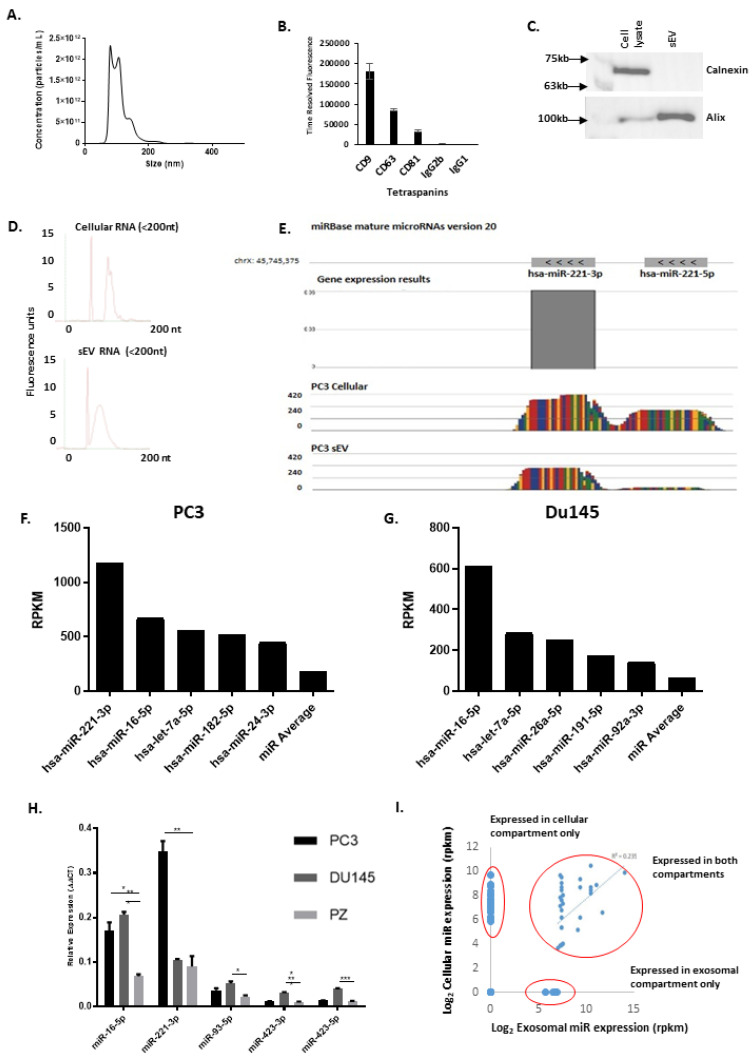
**Extracellular Vesicle Characterisation and miR Cargo Identification**. (**A**), Size distribution of isolated vesicles. sEVs isolated from PC3 cell conditioned medium using a sucrose cushion centrifugation, then analysed by nanosight NTA. (**B**), Immuno-phenotyping assay of PC3 sEVs for exosome associated tetraspanins CD9, CD63, and CD81 compared to isotype controls. Graph shows average minus isotype control for triplicate experiments. (**C**), Western blot analysis of exosome characterisation proteins ALIX and Calnexin from PC3 whole cell lysates and PC3 sEV lysate. (**D**), RNA bioanalysis of the <200 nt fraction from cellular and EV total RNA. (**E**), Mir-Seq mapping alignment analysis of a representative miR (miR-221) from the sequenced miR library from PC3 cells. Data is plotted using Partek Genomics Suite software, using miRBase version 20, and the human genome (hg19) as a reference. Colours indicate DNA bases. Graph height represents normalised read depth. (**F**,**G**), MiR expression profile of the 6 highest expressed miRs from PC3 (**F**) and Du145 (**G**) EV samples. Data is normalised via the RPKM method, using Partek Genomics Suite software. (**H**), Q-PCR validation analysis of miR expression from PC3 and Du145 PCa cells lines compared to the Z-HPV-7 normal prostate immortalised cell line. (**I**), correlation analysis of the miR expressed in cellular and EV compartments in PC3 cells. * *p* = <0.05, ** *p* = <0.01, *** *p* = <0.001.

Since we showed that the microRNA could be EV-associated, we produced purified sEVs from large bioreactor flasks of PC3 cells. Firstly, size distribution was analysed by NanoSight analysis, revealing a <200 nm population, (see Figure 4A). Immobilised vesicles enabled immunostaining of their surface, which demonstrated the expression of several tetraspanin proteins as expected (see Figure 4B). Western blotting on cell versus EV lysates showed a good vesicle separation and purity, as determined by Alix (an endosome-related protein), and Calnexin (ER-associated), as shown in Figure 4C, and Supplementary Figure S11).

Ultracentrifugation to deplete sEVs was successful (see Supplementary Figure S3A) and the pelleted vesicles were resuspended and measured again at 100 nm approx. (see Supplementary Figure S3B). This agreed with the miR levels seen in ultra-centrifuged conditioned media, as seen in Figure 3D. When the EVs were treated with RNase (or mock treated) and then analysed for microRNA content, no significant difference was seen, indicating that miRs were protected from nuclease destruction, consistent with their location intraluminally within vesicles and not internal and protected and not non-specifically associated with the outside of sEVs. (see Supplementary Figure S4).

We then went on to examine PC3 cell and sEVs miR cargos and compare the RNA repertoire of cells to EVs—as other studies showed a poor correlation. RNA was extracted and size-fractionated. The <200 bp fraction was analysed (see Figure 4D) and showed a clear peak for <200 bp RNA species, and an absence of rRNA peaks. A library of small RNA was then sequenced. Figure 4E shows the alignment of reads from the sequencer mapped to the chromosomal location of miR-221-3p on chromosome X, as an example.

The highest expressed miRs in PC3 sEVs are shown in Figure 4F. MiR-221-3p was found to be the highest expressed miR in PC3 sEVs (Figure 4F). Du145 cells and EVs, showed similar results (see Figure 4G). Other miRs highly expressed in both PCa cell lines’ EVs included *hsa*-miR-let-7a-5p and *hsa*-miR-16-5p. Mir-16, 221 were expressed at very high levels in PCa cells compared with cells derived from normal prostate tissue (PZ-HPV7), upon qPCR validation, as shown in Figure 4H. MiR-221 and miR-16 could be detected at high levels in the conditioned media from PC3 cells (see Supplementary Figure S5A,B). Interestingly, the cellular miR populations of PC3 and their associated sEVs were not correlative (see Figure 4I). Additionally, it could be seen that in addition to differences in expression between mature cellular and EV miR species, there were differences in 3 p to 5 p ratios and more pre-miR RNA strands were evident in cellular RNA (see Figure 4E).

### 3.5. EV from PC3 Cells Induce Mineralisation of 7F2 Osteoblast Cells

**Figure 5:**
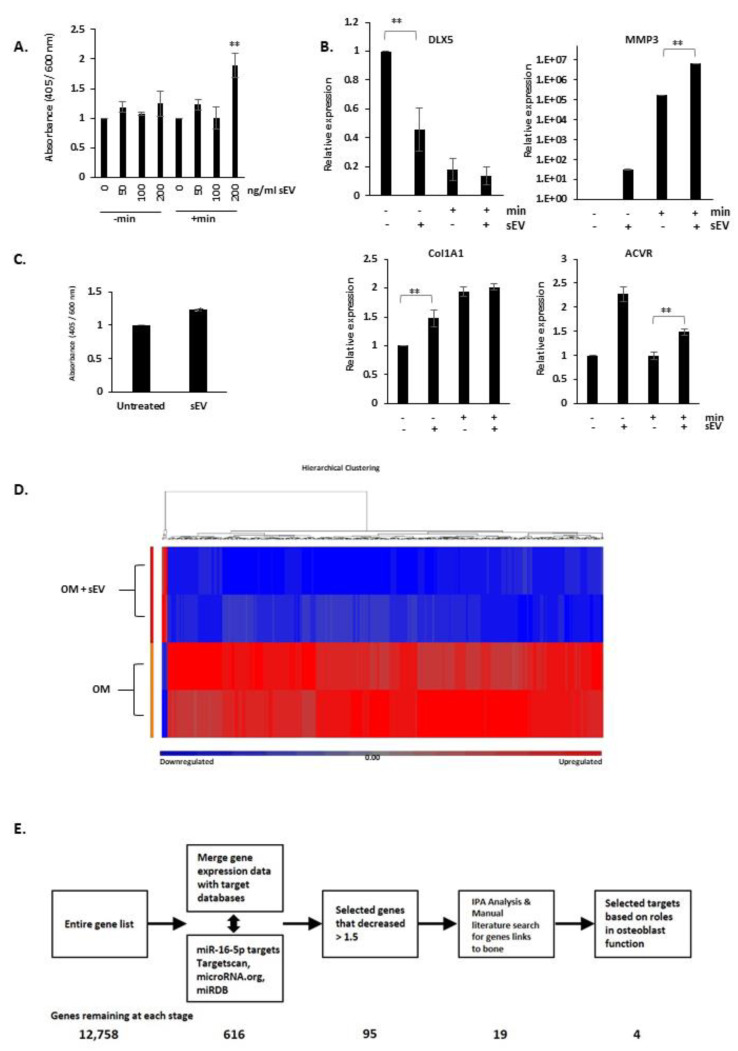
**EVs from PC3 cells induce mineralisation of 7F2 osteoblasts.** Mineralisation of sEV treated 7F2 cells. (**A**), Mineralisation of 7F2 cells treated with 0–200 ug/mL sEVs for 10 days with (+min) or without osteogenic media (-min). Graph shows alizarin red absorbance (405 nm) normalised to a post stain crystal violet stain (600 nm) for cell number. (**B**), Q-PCR analysis of osteoblast differentiation genes in 7F2 cells treated with sEVs (+) or with PBS vehicle (-) in regular (-) or with osteogenic media (+). Data is normalised to housekeeping genes β-actin, GAPDH and RPL19. (**C**), Mineralisation of 7F2 cells treated with 200 ug/mL sEVs for 10 days (or PBS control) in the presence of β-glycerophosphate. Graph shows alizarin red absorbance (405 nm) normalised to a post stain crystal violet stain (600 nm) for cell number. (**D**), Hierarchical clusteringdata from RNA-seq of 7F2 cells treated with sEVs. Data shows genes significantly changed in 7F2 by sEV treatment. Data represents two independent experiments. (**E**), Flow chart schematic of the data analysis pathway and miR target prediction, followed by Ingenuity pathway analysis and manual searches for osteoblast activity. ** *p* = <0.01.

Next, we treated mineralising 7F2 cells with PC3 sEVs (0–400 ng/mL). Below 200 ng/mL did not produce significant effects on mineralisation but at 200 ng/mL, EVs stimulated 7F2 mineralisation (see Figure 5A).

We then examined whether sEV treatments could affect mineralisation gene expression. Treating 7F2 cells with osteogenic media and EVs (200 ng/mL) increased the expression of *MMP3*, *ACVR1* and *Col1A1*. EV treatment reduced *DLX5* expression (see Figure 5B). Interestingly, in the absence of mineralising hormones, these gene expression changes could also be seen, however, without the source of phosphate (β-glycerophosphate) the EVs could not induce mineralisation (see Figure 5C). With phosphate, EV treatment alone could induce minimal mineralisation.

We then examined the effects of EV treatment on the global gene expression of 7F2 cells. 7F2 cells were mineralised for 48 h ± EVs (200 ng/mL), and the mRNA constituent subjected to RNA-seq. Figure 5D shows the hierarchical clustering of the gene expression found, showing that 470 statistically significant genes changed by 1.5 ± fold, and only six showed upregulation, whereas 464 showed downregulation.

Bioinformatic analysis showed pathway enrichment in “connective tissue development and function” and “skeletal and muscular system development and function” (see Supplementary Figure S6A). Upstream effector prediction showed several miRs were significant (see Supplementary Figure S6B). One miR was miR-16-5p, highly expressed in both PC3 cells and PC3 sEVs, and was the highest in Du145 sEVs. Analysis of miR-16-5p using the TCGA Prostate Cancer (PRAD) database showed higher levels of miR-16-5p correlated with lowered overall survival in PCa patients (*n* = 397) (see Supplementary Figure S7). Other upstream regulator miRs showed a similar pattern, but with lowered p values (see Supplementary Figure S8). Since bone metastasis is correlated with poor overall survival, we decided to analyse miR-16-5p further, to analyse its influence on osteoblast mineralisation, in our model.

To analyse potential miR-16-5p targets involved in bone mineralisation, we performed a bioinformatic search. From the genes with changed expression in sEV-treated 7F2 cells, targets were selected in a stepwise manner. The 12,000 expressed genes were cross-referenced with predicted miR-16-5p targets from three independent databases (Targetscan, miRDB and microrna.org), to generate a list of 616 predicted targets. Genes whose expression changed <1.5 ± fold were removed, leaving 95, of which 19 were found to have links to bone related studies, by the literature analysis. From this list of genes, four were found to be linked to osteoblast function, and were selected as potential mediators (see Figure 5E and Supplementary Figure S6C) and were further studied as “proof of principle”.

### 3.6. The Effects of miR-16 on Osteoblasts

**Figure 6:**
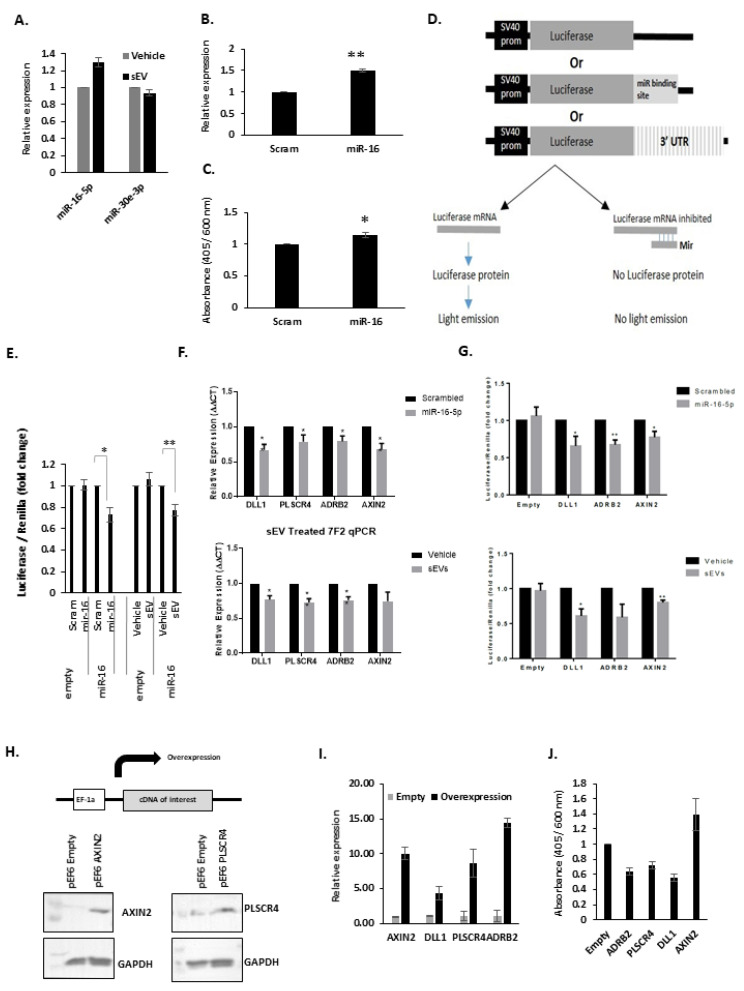
**The effects of miR-16 in 7F2 osteoblasts**. (**A**), Q-PCR analysis of miR-16 and miR-30 expression in sEV treated 7F2 cells. Data is normalised to U18 small nuclear RNA. (**B**), Q-PCR analysis of miR-16 expression in 7F2 cells transfected with either scrambled oligo or miR-16 mimic (5 pmol). Data is normalised to U18 small nuclear RNA. (**C**), Alizarin red mineralisation assay on 7F2 cells transfected with either scrambled oligo or miR-16 mimic (5 pmol). Graph shows alizarin red absorbance (405 nm) normalised to a post stain crystal violet stain (600 nm) for cell number. (**D**), schematic diagram of the luciferase empty, miR-reporter or UTR reporter plasmid constructs, and the downstream effects of miRs on their activity. (**E**), Luciferase assays on 7F2 cells transfected with either empty or miR16 reporter luciferase constructs and either scrambled oligo or miR-16 mimic. Data is normalised to the internal constitutively expressed renilla luciferase activity. (**F**), Upper panel-Q-PCR analysis of the expression of the four miR-16 gene targets in 7F2 cells transfected with either scrambled or miR-16 mimic oligos. Lower panel-Q-PCR analysis of the expression of the four miR-16 gene targets in 7F2 cells treated with either sEVs or PBS control. Data is normalised to housekeeping genes -actin, GAPDH and RPL19. (**G**), Upper panel-Luciferase UTR reporter assays of the four miR-16 gene targets in 7F2 cells transfected with either scrambled or miR-16 mimic oligos. Lower panel-Luciferase UTR reporter assays of the expression of the four miR-16 gene targets in 7F2 cells treated with either sEVs or PBS control. Data is normalised to the internal constitutively expressed renilla luciferase activity. (**H**), Upper panel, schematic diagram of the overexpression plasmid constructs (pEF6) for overexpression of the four miR-16 target genes. Lower panel–Western blot for Axin2 and PLSCR4 in 7F2 cells transfected with pEF6-Axin2 and pEF6-PLSCR4 respectively. Protein was normalised to GAPDH levels. (**I**), Q-PCR expression analysis of the four miR-16 targets in transfected 7F2 cells. Data is normalised to housekeeping genes β-actin, GAPDH and RPL19. (**J**), Alizarin red staining of 7F2 cells stably transfected with either pEF6 empty or pEF6 expressing Axin2, ADBR2, PLSCR4 or DLL1. Graph shows alizarin red absorbance (405 nm) normalised to a post stain crystal violet stain (600 nm) for cell number. * *p* = <0.05, ** *p* = <0.01.

To confirm whether miR-16-5p levels increase in recipient cells following sEV treatment, 7F2 cells were treated with sEV (200 ug/mL) for 8 h, washed thoroughly and RNA was collected. Treatment with sEVs increased (30%) the miR-16 expression. MiR-30e-3p, which is not detectable in EVs, did not change (see Figure 6A). MiR-16-5p and miR-30e pre-miR levels did not change. A miR-16 mimic oligo could also be introduced by lipid-mediated transfection and detected by qPCR (see Figure 6B) and could increase mineralisation (see Figure 6C). MiR-16-5p may, therefore, be involved in pro-osteoblastic sEV communication between PC3 and 7F2 cells, and that miR-16-5p is likely transferred by PC3 EVs to 7F2 cells. We, therefore, looked for changes in miR-16-5p mRNA targets in sEV-treated 7F2 cells that may explain the increased mineralisation.

To monitor miR-16-5p activity, we constructed a miR-16 reporter (and empty vector control) (see Figure 6D), which were used to stably transfect 7F2 cells. Cells containing either the miR-16-5p reporter, (or empty vector), were then transfected with scrambled or miR-16–5*p* mimic oligos. In miR-16-5p reporter 7F2 cells, transfection of miR-16-5p oligo resulted in a significant decrease in luciferase activity compared to the scrambled oligo (*p* = 0.015) (Figure 6E). miR-16-5p transfection had no effect on luciferase activity in empty reporter 7F2 cells versus scrambled.

7F2 miR-16-5p reporter cell lines (and empty) were treated with PC3 sEVs (200 μg/mL) or vehicle (PBS). In empty reporter 7F2 cells, sEV treatment had no effect on luciferase activity compared to vehicle treatment (Figure 6E). In 7F2 miR-16-5p reporter cells, sEVs treatment significantly reduced luciferase activity by 23% (*p* = 0.01). This is consistent with data showing a rise in miR-16-5p levels with sEV treatment.

To confirm miR-16-5p modulation, 7F2 cells were transfected with 25 pmol/mL of either a scrambled or miR-16-5p, and the expression of the four genes was analysed by qPCR (Figure 6F upper panel). MiR-16-5p transfection significantly decreased the expression of all four genes compared to scrambled oligo.

To test whether sEV-induced cellular increase in miR-16-5p is sufficient to modulate these four genes, 7F2 cells were treated with 200 μg/mL of sEVs or vehicle (PBS) and gene expression was analysed by qPCR. The expression of all four genes was reduced in sEV treated cells compared to controls by 23%–27%, see Figure 6F, lower panel. TargetScan analysis confirmed the complementary miR-16-5p sites in the four selected targets, with two sites in the DLL gene (see Supplementary Figure S9).

The qPCR of sEV-treated 7F2 cells highlights the modulation of gene expression seen in these cells. However, qPCR alone is unable to address the cellular mechanisms by which these regulatory effects may occur. To aid this, luciferase reporters were utilised. Mouse miTarget^TM^ 3′UTR reporters were purchased from GeneCopoedia (no *Mus musculus* PLSCR4 plasmid was available at the time of the study) and were transiently co-transfected with either scrambled or miR-16-5p oligos or with PC3 sEVs. Transiently transfected empty plasmid was used as the control. When co-transfected with miR-16-5p, there was a significant reduction in the relative luminescence from the DLL1, ADRB2 and AXIN2 reporters compared to the scrambled oligo (*p* < 0.05 for all) (Figure 6G upper panel). When co-treated with sEVs, a similar reduction in activity was seen (Figure 6G lower panel).

### 3.7. Overexpression of miR-16-5p Targets

Plasmids expressing the cDNA for these genes were synthesised, and stably transfected into 7F2 cells to test the effect of the overexpression of these miR-16-5p targets. Overexpression was validated by qPCR and had no effect on 7F2 cell growth.

Western blotting confirmed Axin2 and PLSCR4 protein overexpression (functional antibodies for DLL1 and ADRB2 were not available). 7F2 cells overexpressing these genes were mineralised alongside 7F2-pEF6-Empty cells for 10 days. ADRB2, PLSCR4 and DLL1 ectopic overexpression resulted in a decrease in mineralisation relative to control cells (*p* < 0.05 for PLSCR4, *p* < 0.01 for ADRB2 and DLL1). Conversely, AXIN2 overexpression resulted in increased mineralisation, although this increase failed to reach significance (*p* = 0.22), see Figure 6J.

## 4. Discussion

Bone is a dynamic tissue, turned over and remodelled by the balanced effects of osteoblasts and osteoclasts. PCa cells influence bone and cause disruption, leading to osteoblastic and rarely osteolytic lesions. The predominance of bone PCa metastasis is by far the most worrying aspect of prostate cancer management, with high morbidity and mortality.

PCa cells release signalling molecules which affect bone cells, with the effects of proteins and cytokines being relatively well known, e.g., TGF-β, BMPs, RANKL (Receptor activator of nuclear factor kappa-Β ligand) and EGF. MiR effects are less well understood. Here, we investigated whether the secreted microRNAs from PCa cells could have an effect on mineralising bone osteoblasts. 7F2 cells showed reproducible, rapid and timely differentiation, culminating in detectable deposits of hydroxyapatite. Key osteoblastic genes were also measured in parallel.

Historically, PC3 cells have been shown to cause primarily osteolytic lesions in mice, with some PC3 subclones forming mixed lesions (19) whereas our results show a strong in vitro osteoblastic phenotype, which could be conflicting. However, our spheroid and co-culture experiments provide evidence that PC3 cells can stimulate localised osteoblastic effects via secreted molecules, and showed a more mixed phenotype when in direct contact, e.g., with spheroid co-cultures, a ring of mineralisation effect was seen surrounding the spheroid, whilst directly underneath, reduced and disrupted mineralisation was seen. Disruption was observed when 7F2 cells were not in mineralising media—indicating a context-dependent PC3 effect. This upholds the observation that PC3 cells can induce both osteolytic and osteoblastic lesions, but in the simple in vitro model used here, there are no osteoclast cells to be stimulated. Additionally, the secreted proteases of PC3 would not be co-purified with EVs. Others have also noted the ability of PC3 cells to stimulate osteoblasts in culture [12,20]. In vivo*,* increased osteoclast activity may outweigh osteoblast activity. However, it is interesting to note that PC3 do have a strongly stimulating effect on mineralisation, similarly to other PCa cell lines—although we acknowledge that in a mature bone environment as observed in a PCa patient, it may be the osteocytes rather than osteoblasts which may show the greater response, and that for PC3 bone metastasis, osteoclastic stimulation may outweigh the osteoblastic effect.

Chromatography and ultracentrifugation experiments concluded that PCa cells secreted large quantities of microRNAs, both free and within EVs which both stimulated osteoblast mineralisation. EV transfer from prostate cancer cells to recipient cells has been shown by our authors, Roberts-Dalton et al., 2017 [22].

MiR-seq of EV contents revealed that several microRNAs were present. MiR-221 was seen to be highest in PC3 EVs, a miR previously found to be essential for the maintenance of PC3 cell mesenchymal transition phenotype and cellular adhesion [23]. Mapping analysis of the read sequences between cellular and vesicular miRs revealed different levels of microRNAs from cellular to vesicular compartments, but also strand ratio differences between 3p to 5p microRNAs. This indicates that miR populations in EVs are not a random sampling of cellular constituents—implying a selective packaging or exclusion mechanism of miRs into the EVs. This is a phenomenon reported by others and the machinery for selection and enrichment are being deciphered [24].

Treating 7F2 with sEVs increased mineralisation and changed marker genes, e.g., DLX5, MMP3 and ACVR. EVs also increased mineralisation without osteogenic media, but in the absence of inorganic phosphate, the effect was minimal. RNAseq on sEV-treated mineralising 7F2 cells revealed gene expression changes. Pathway analysis found “skeletal development” to be the main correlated pathways. Upstream analysis indicated several proteins of interest which could be responsible including TGF-β, Notch and Wnt, however, we were interested in the possibility of miRs as upstream regulators. Several miRs were implicated as upstream modulators including miRs-143, 1–3p, 21, 199, 140 and miR-16–5p. They all had lowered survival in PCa patients (see Supplementary Figure S8).

The effects of PC3 EVs on osteoclasts were also studied. Interestingly, although EVs increased the expression of osteoclast differentiation genes in the presence of RANKL, EV treatment inhibited osteoclast formation and function in a pit formation assay. This would again indicate that PCa-derived EVs may have a role to play in the reducing osteoclasts and increasing osteoblast activity, (see Supplementary Figure S10).

Mir-16-5p was the highest expressed miR in Du145 cells and the second highest in PC3 cell EVs, it was present in both cellular and EVs compartments and was implicated as an upstream regulator in the IPA analysis, therefore we decided to investigate it further. MiR-16 increased in EV treated 7F2 cells, however, pre-miR-16 levels did not change, indicating that the mature miR-16-5p was exogenous. EV treatment and miR-16 mimic transfection both increased 7F2 mineralisation.

We saw reduced activity of miR-16 luciferase reporters both with transfected miR-16-mimic and when treated with EVs, indicating that EV treatment “supplied” 7F2 cells with biologically active miR-16.

Four representative miR-16-5p targets were studied in EVs treated 7F2 cells-DLL-1, AXIN2, ADBR2 and PLSCR4. DLL-1 is a ligand involved in the Notch receptor signalling cascade, with a complex and unclear role in bone development. Embryonic deletion is lethal in mice, whereas haploinsufficiency studies revealed several phenotypes including reduced lumbar vertebrae, kinked tail and bone density reduction, which would contradict our study. However, DLL-1 seems to have two very different activities in bone; *i*, positive regulation of immature osteoblast differentiation and expansion, but *ii*, a negative regulator of functional osteoblast maturation. Since our studies involve the functional and terminal differentiation of osteoblasts, then the reduction of DLL-1 via miR-16-5p may increase the maturation of differentiated osteoblasts, i.e., mineralisation.

AXIN2 (Axis inhibition protein 2) makes up a complex which degrades β-catenin, negatively regulating Wnt signalling. Axin2^KO^ mice have increased osteoblast proliferation and differentiation, and a reduced osteoclast activity [25]. This agrees with the reduction of Axin2 in the mouse osteoblasts treated with EVs or miR-16-5p mimic, which showed increased mineralisation.

Similarly, ADBR2 (β2-adrenergic receptor) is expressed on various bone cell types, and evidence indicates it plays a role in bone remodelling, and receptor stimulation inhibits osteoblastic bone formation and enhances osteoclastic bone resorption [26,27]. *Adbr2^KO^* mice demonstrate an increased bone mass [28].

The dissipation of membrane lipid asymmetry in response to increased Ca^2+^ is a critical mechanism for cellular events, e.g., coagulation, bone mineralization, and cell–cell interactions. Scramblases such as PLSCR4 induce non-specific trans-bilayer movements, but have conflicting evidences in bone. Kubista et al. showed that PLSCR4 was downregulated in osteoblastic vs. non-osteoblastic osteosarcomas [29], whereas Andersen et al. showed PLSCR4 to be upregulated in high bone-forming hBMSC cells [30].

## 5. Conclusions

PCa cells promote the mineralisation of 7F2 osteoblasts in vitro, either via direct contact or via secreted factors including EVs. PCa cells secrete a large quantity of miRs both as free oligos or associated with EVs. We show that miRs, present in PC3 EVs, can also affect recipient cells, and can influence a small cohort of genes selected as miR-16-5p targets involved in osteoblastic function. MiR-16 downregulates genes that inhibit or reduce mineralisation and osteoblast differentiation. PCa-produced EVs may influence local bone cells at sites of metastasis and may play a part in prostate cancer bone osteoblastic lesion formation.

## Data Availability

The data presented in this study are available in supplementary material.

## Supplementary Materials

The following are available online at https://www.mdpi.com/article/10.3390/biology10040318/s1. Figure S1. A, Q-PCR analysis of miRNA-132 expression from a panel of prostate cell lines, normalised to U18 snRNA and plotted relative to the RWPE-1. B, Q-PCR analysis of miR-132 release into conditioned media form PC3 cells (48hrs), normalised to U18 snRNA. C, Q-PCR analysis of miR-132 from RNA ex-tracted from media and conditioned media after being subjected to increasing forces of centrifugation. D, Q-PCR analysis of miR-27a, and miR-21 from media, and conditioned media from Du145 wt and Du145 Rab27ko cells over 48hours. Data is normalised to U18 snRNA. E, Q-PCR analysis for miR-27a, from RNA ex-tracted from conditioned media from Du145wt and Du145 Rab27ko cells subjected to size exclusion chroma-tography, over 30 × 500 uL fractions. Data was normalised to a C. elegans miR-39 spike-in oligo. F&G, Q-PCR analysis of miR-27a from RNA extracted from DMEM spiked with EVs and and C. elegans RNA and subjected to size exclusion chromatography, over 30 × 500 uL fractions. Data was normalised to a C. el-egans miR-39 spike-in oligo. H, Protein quantification analysis of each fraction from size exclusion chro-matography of conditioned medium from PC3 cells.* *p* = < 0.05, ** *p* = < 0.01, *** *p* = < 0.001. Figure S2. MiR-27a RNA oligo was purchased from MWG-Eurofin, and was serially diluted in DMEM (500 mL) to create a range from 0.3mM to 7 × 10−8 mM. RNA was then extracted using Trizol-LS, and subjected to RT-PCR for miR-27a. This was used to generate a standard curve for miR-27a levels. PC3 conditioned media was similarly ex-tracted and Ct values compared and measured. Figure S3. Size distribution of isolated EVs from PC3 cell conditioned media, as analysed by Nanosight NTA. A. EV analysis from conditioned media before and after centrifugation to deplete EVs. B. Analysis of the EV particle size from the resuspended pellet from EV centrifugation from conditioned media. Figure S4. Bioanalyser analysis of RNase treated PC3 EVs. RNA was extracted from EVs mock treated or treated with RNase and assessed using a Bioanalyser. Figure S5. A. QPCR analysis of the highest miRs found in PC3 EVs. miR-221 and miR-16 from PC3 conditioned media. Mir-222 is given as a low expression miR for comparison. B. QPCR analysis of miR-16 found in PC3 conditioned media. Figure S6. A. Bioinformatic analysis and pathway enrichment using the Ingenuity Pathway Analysis software. Data taken from 7F2 cells treated with osteogenic media and 200 ng/mL EVs. B. Upstream microRNA effector analysis prediction using Ingenuity Pathway analysis. Using gene expression data from 7F2 cells treated with osteogenic media and 200 ng/ml EVs. C. Four gene targets of miR-16-5p chosen as a proof of principle to follow the effects of EVs on the mineralisation of 7F2 cells. Figure S7. Overall survival probability data and copy number of miR-16-5p in prostate cancer cases. A. Kaplan Meier survival data for miR-16-5p. Mir levels are higher in patients with a lower related overall survival prob-ability. Data from the TCGA Prostate Cancer (PRAD) database, n = 397. *p* = 0.0086. B. Copy number of miR-16-5p is higher in patients with metastatic prostate cancer compared to normal. Data from the TCGA Prostate Cancer (PRAD) database n = 502. Figure S8. Kaplan Meier survival data for A, miR-140, B, miR-143, and C, miR-199. Mir levels are higher in patients with a lower related overall survival probability. Data from the TCGA Prostate Cancer (PRAD) database, n = 397. *p* = 0.0086. Figure S9. Confirmation of the miR-16 complementarity of the miR-16 selected targets—including A. DLL1, B. Axin2, C. PLSCR4, and D. ADBR2. Note DLL1 has two miR-16 binding sites. Figure S10. EVs Stimulate Osteoclast Differentiation but Reduce Osteoclast Activity. Morphology of RAW Cells. A. Microscopy images of TRAP stained RAW cells grown on 6 well plates for 8 days in either DMEM or B. DMEM + 25 ng/mL RANKL; Scale Bar: 250 μm. C. Q-PCR expression analysis of osteoclast differentiation genes OSCAR and TRAP in RAW cells treated with RANKL (0–25 ng/mL). Graphs show average fold change compared to control +SEM, n = 3. Statistical significance assessed by one-way ANOVA, * *p* < 0.05, ** *p* < 0.01, *** *p* < 0.001. D. Q-PCR expression analysis of osteoclast differentiation genes OSCAR and TRAP in RAW cells treated with RANKL/sEVs. (25ng/mL) (R) and 200 μg/mL of sEVs. Graphs show average fold change compared to control +SEM, n = 3. Statistical significance assessed by unpaired test, * *p* < 0.05, ** *p* < 0.01. E–G. Pit formation assay on RANKL and RAW cells treated treated with 25ng/ml of RANKL and 200ng/ml of sEVs. E. Control and sEV treated cells in the absence of RANKL. F. Control and sEV treated cells with RANKL. G. Average resorbed area of control and sEV treated cells (-E/+E) with RANKL (R). Graphs show average +SEM, n = 3. Statistical significance assessed by unpaired ttest. * *p* < 0.05. Figure S11. Characterisation of PC3 sEV protein. Western blot of exosome characterisation proteins; ALIX and Calnexin from PC3 whole cell lysate (CL) (20 μg) and PC3 sEV lysate (EXO) (20 μg). Densitometry analysis is given underneath. Figure S12. Higher magnification images of the mineralising 7F2 cells, stained with alizarin red. Text S1. Supplemental materials. Polymerase Chain Reaction. Western Blotting. sEV Isolation. Bioreactor Flask Setup. Media Collection. sEV Validation
