# Supplementary material for: Prostate Cancer Cell Extracellular Vesicles Increase Mineralisation of Bone Osteoblast Precursor Cells in an In Vitro Model
Source: Biology (Basel). 2021 Apr 10;10(4):318. doi: 10.3390/biology10040318 (PMC8069461; doi:10.3390/biology10040318)
Supplement: Supplementary file 1 [file biology-10-00318-s001.zip › biology-1126868-supplementary/biology-1126868-supplementary-for conversion.docx]

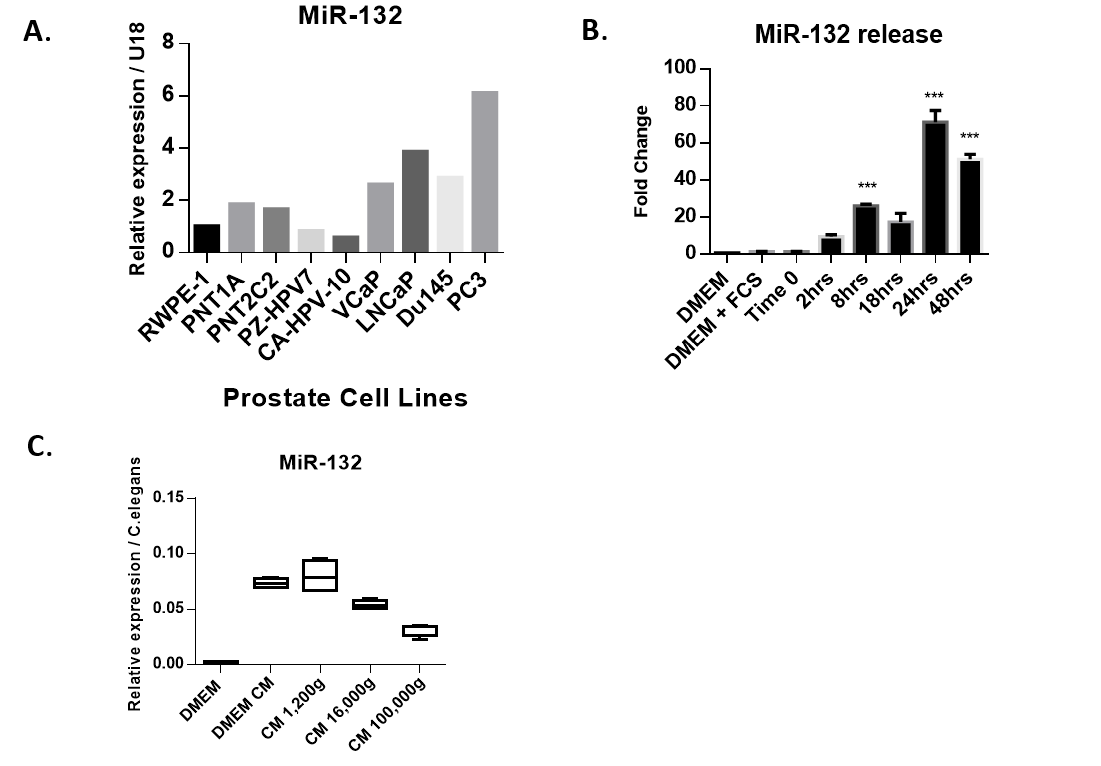


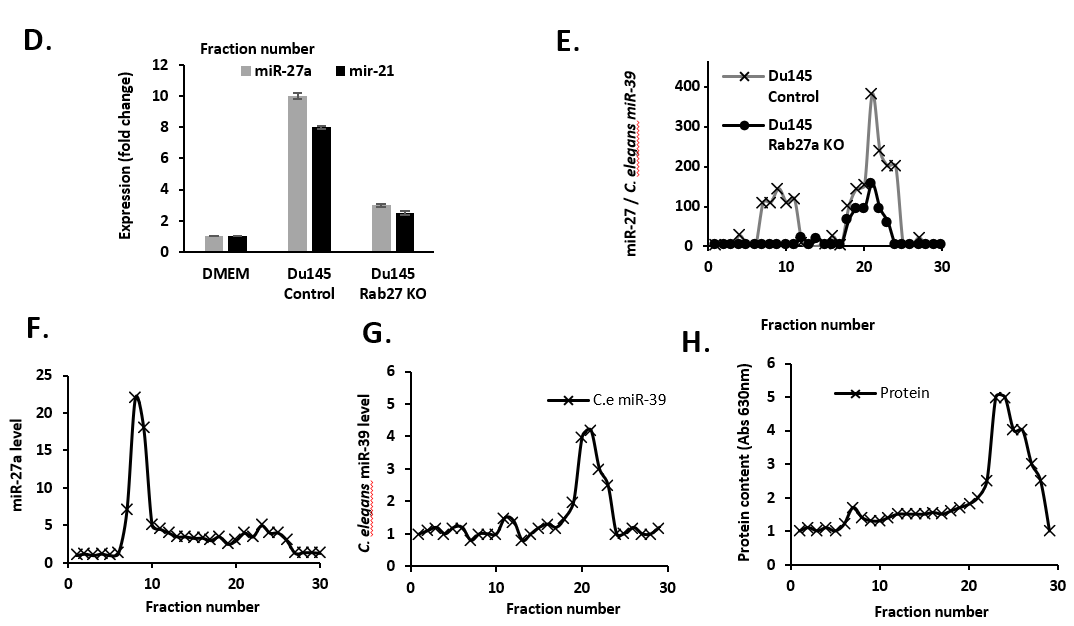


**Figure S1. A**, Q-PCR analysis of miRNA-132 expression from a panel of prostate cell lines, normalised to U18 snRNA and plotted relative to the RWPE-1. **B**, Q-PCR analysis of miR-132 release into conditioned media form PC3 cells (48hrs), normalised to U18 snRNA. **C**, Q-PCR analysis of miR-132 from RNA extracted from media and conditioned media after being subjected to increasing forces of centrifugation. **D**, Q-PCR analysis of miR-27a, and miR-21 from media, and conditioned media from Du145 ^wt^ and Du145 ^Rab27ko^ cells over 48hours. Data is normalised to U18 snRNA. **E,** Q-PCR analysis for miR-27a, from RNA extracted from conditioned media from Du145^wt^ and Du145 ^Rab27ko^ cells subjected to size exclusion chromatography, over 30 × 500ul fractions. Data was normalised to a C. elegans miR-39 spike-in oligo. **F&G**, Q-PCR analysis of miR-27a from RNA extracted from DMEM spiked with EVs and and C. elegans RNA and subjected to size exclusion chromatography, over 30 x 500ul fractions. Data was normalised to a C. elegans miR-39 spike-in oligo. **H**, Protein quantification analysis of each fraction from size exclusion chromatography of conditioned medium from PC3 cells.* *p* = < 0.05, ** *p* = < 0.01, *** *p* = < 0.001.


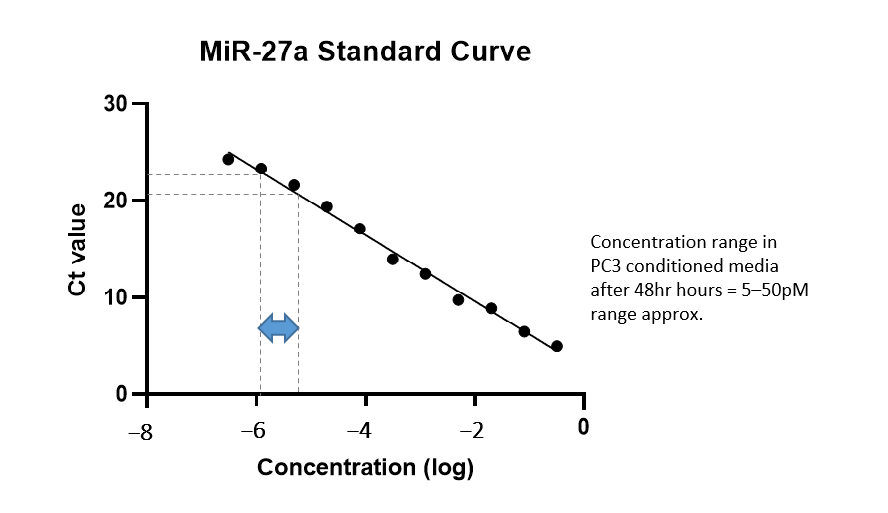


**Figure S2.** MiR-27a RNA oligo was purchased from MWG-Eurofin, and was serially diluted in DMEM (500ml) to create a range from 0.3mM to 7 × 10^−8^mM. RNA was then extracted using Trizol-LS, and subjected to RT-PCR for miR-27a. This was used to generate a standard curve for miR-27a levels. PC3 conditioned media was similarly extracted and Ct values compared and measured.

**A.**

**B.**


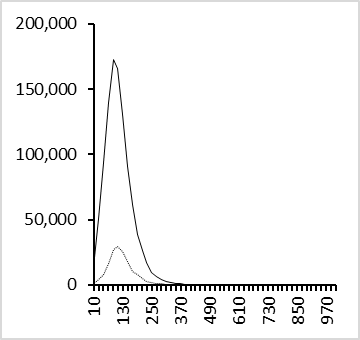

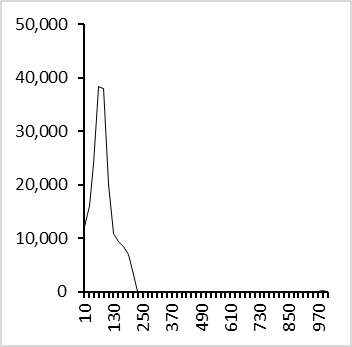


**Particle size (nm)**

**Particle size (nm)**

**Nanoparticle concentration 10^6^/ml)**

**Nanoparticle concentration 10^6^/ml)**

**CM**

**Depleted CM**

**Figure S3.** Size distribution of isolated EVs from PC3 cell conditioned media, as analysed by Nanosight NTA. **A.** EV analysis from conditioned media before and after centrifugation to deplete EVs. **B.** Analysis of the EV particle size from the resuspended pellet from EV centrifugation from conditioned media.


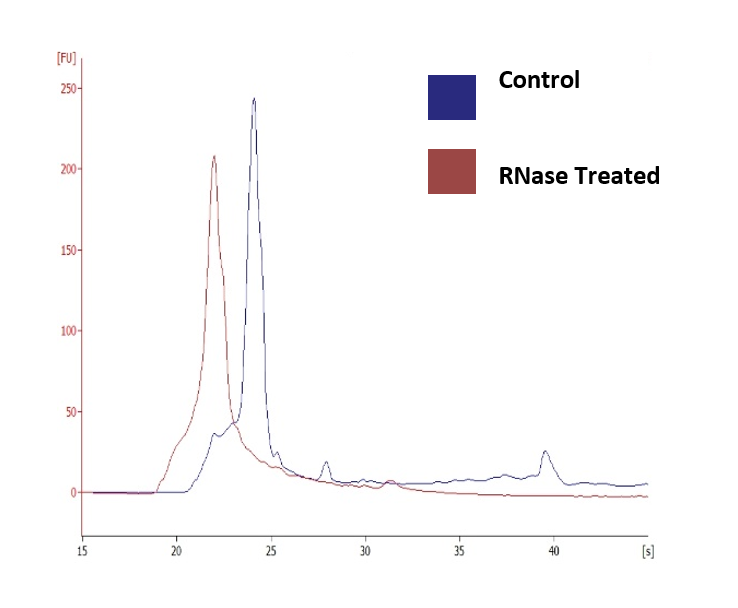


**Figure S4.** Bioanalyser analysis of RNase treated PC3 EVs. RNA was extracted from EVs mock treated or treated with RNase and assessed using a Bioanalyser.


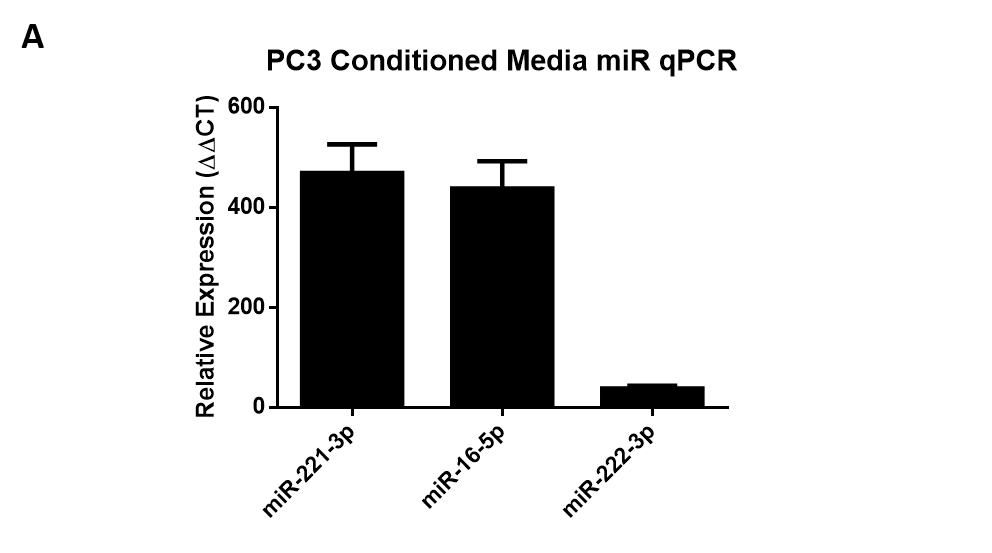


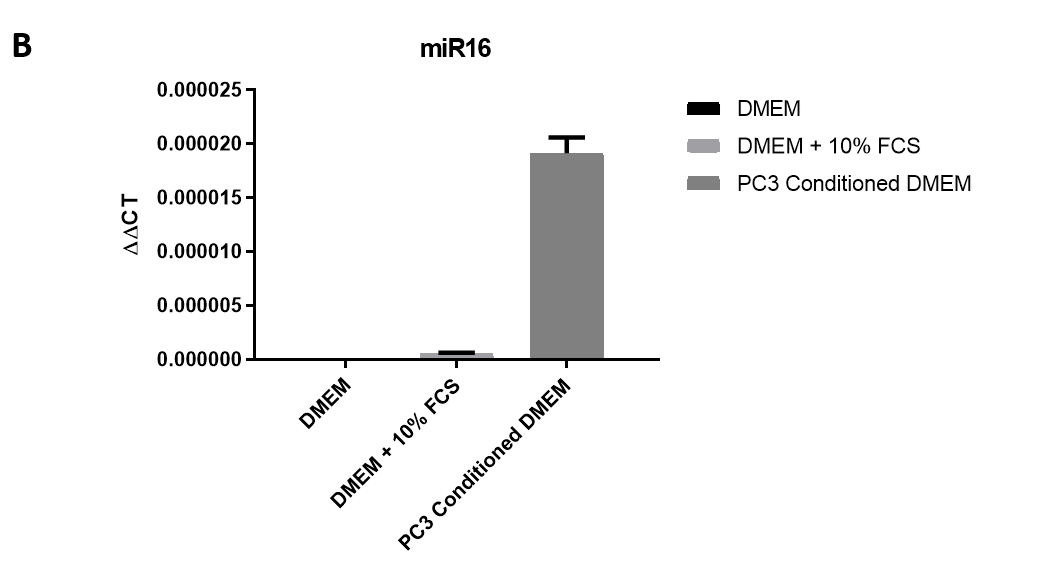


**Figure S5. A.** QPCR analysis of the highest miRs found in PC3 EVs. miR-221 and miR-16 from PC3 conditioned media. Mir-222 is given as a low expression miR for comparison. **B.** QPCR analysis of miR-16 found in PC3 conditioned media.


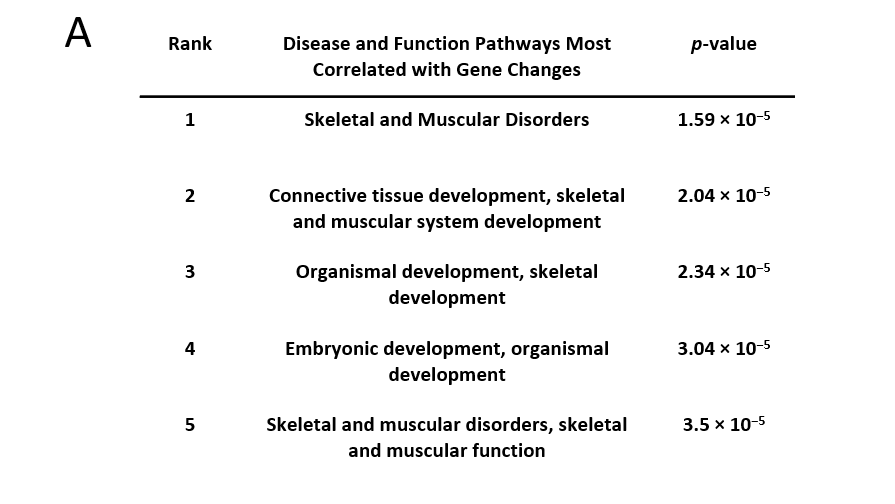


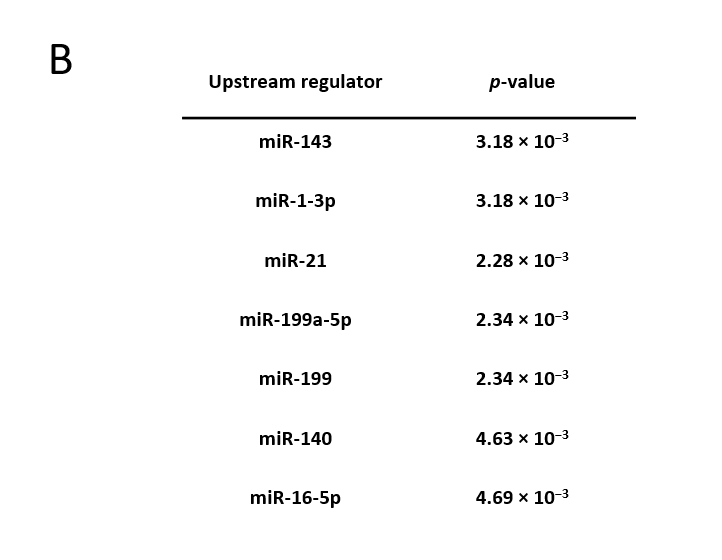


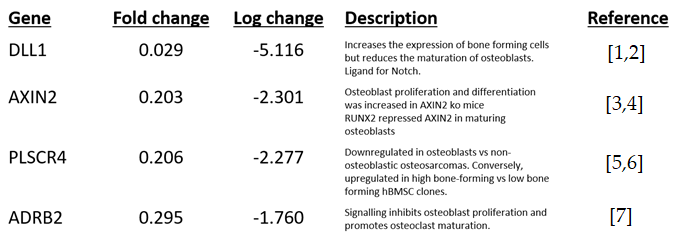


**Figure S6. A.** Bioinformatic analysis and pathway enrichment using the Ingenuity Pathway Analysis software. Data taken from 7F2 cells treated with osteogenic media and 200ng/ml EVs. **B.** Upstream microRNA effector analysis prediction using Ingenuity Pathway analysis. Using gene expression data from 7F2 cells treated with osteogenic media and 200ng/ml EVs. **C.** Four gene targets of miR-16-5p chosen as a proof of principle to follow the effects of EVs on the mineralisation of 7F2 cells.


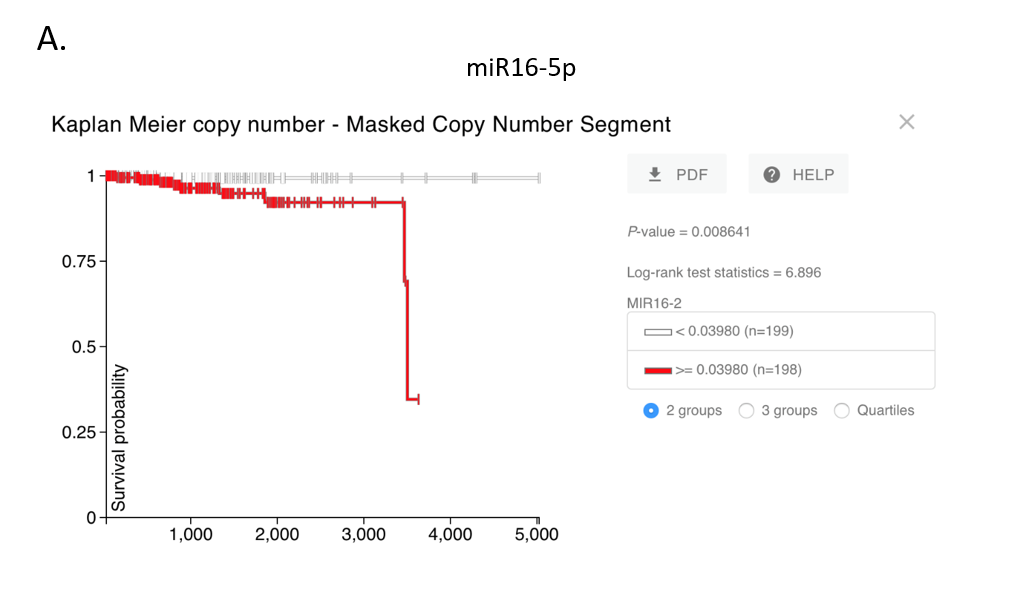


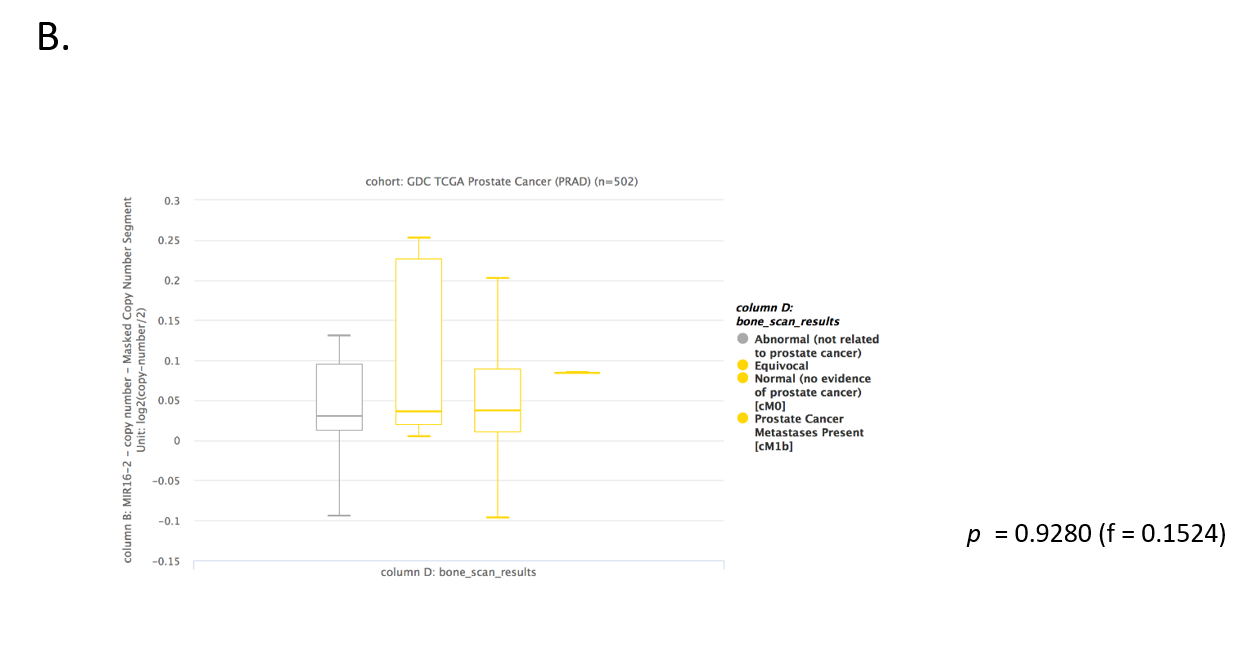


**Figure S7.** Overall survival probability data and copy number of miR-16-5p in prostate cancer cases. **A.** Kaplan Meier survival data for miR-16-5p. Mir levels are higher in patients with a lower related overall survival probability. Data from the TCGA Prostate Cancer (PRAD) database, *n* = 397. *p* = 0.0086. **B.** Copy number of miR-16-5p is higher in patients with metastatic prostate cancer compared to normal. Data from the TCGA Prostate Cancer (PRAD) database *n* = 502.


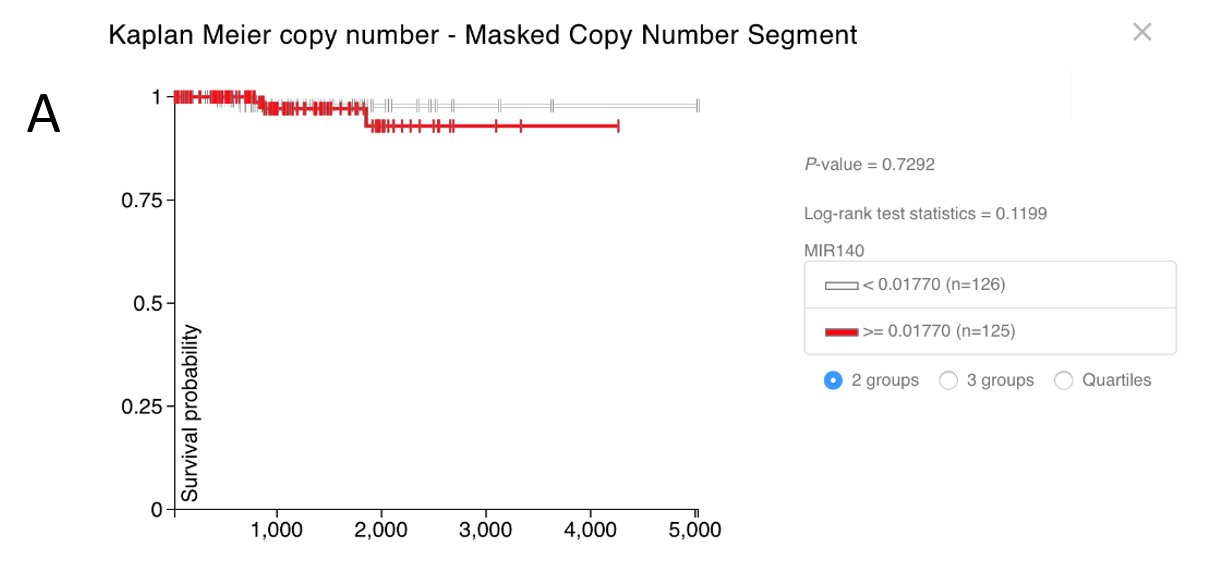


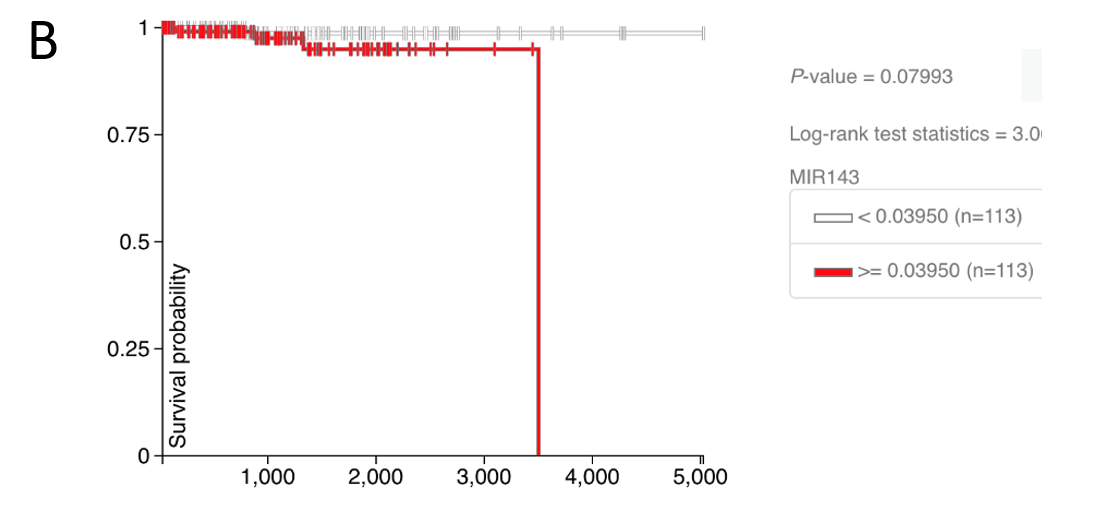


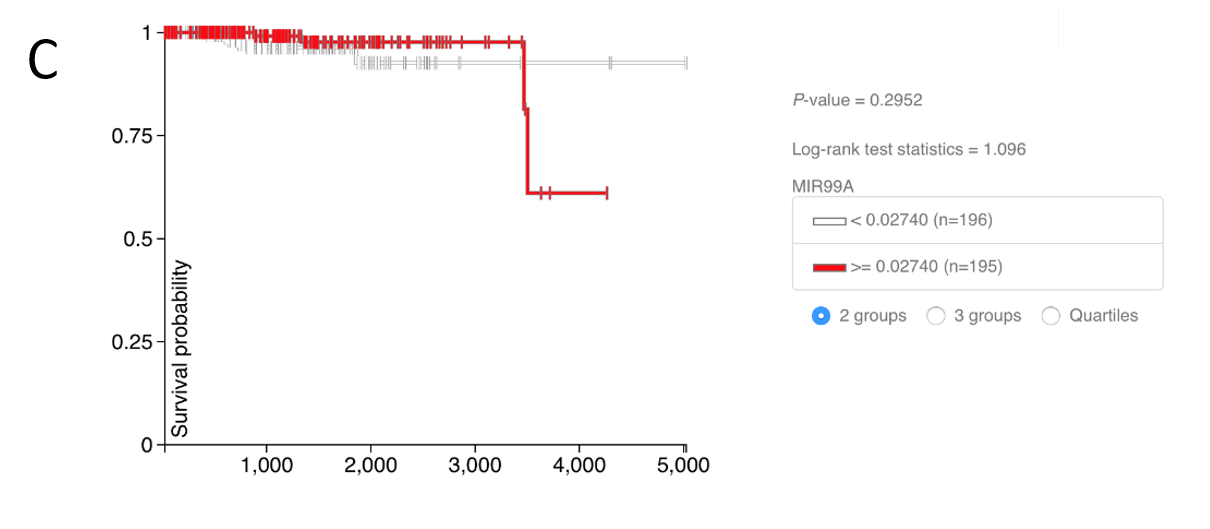


**Figure S8.** Kaplan Meier survival data for **A,** miR-140, **B,** miR-143, and **C,** miR-199. Mir levels are higher in patients with a lower related overall survival probability. Data from the TCGA Prostate Cancer (PRAD) database, n = 397. p = 0.0086.


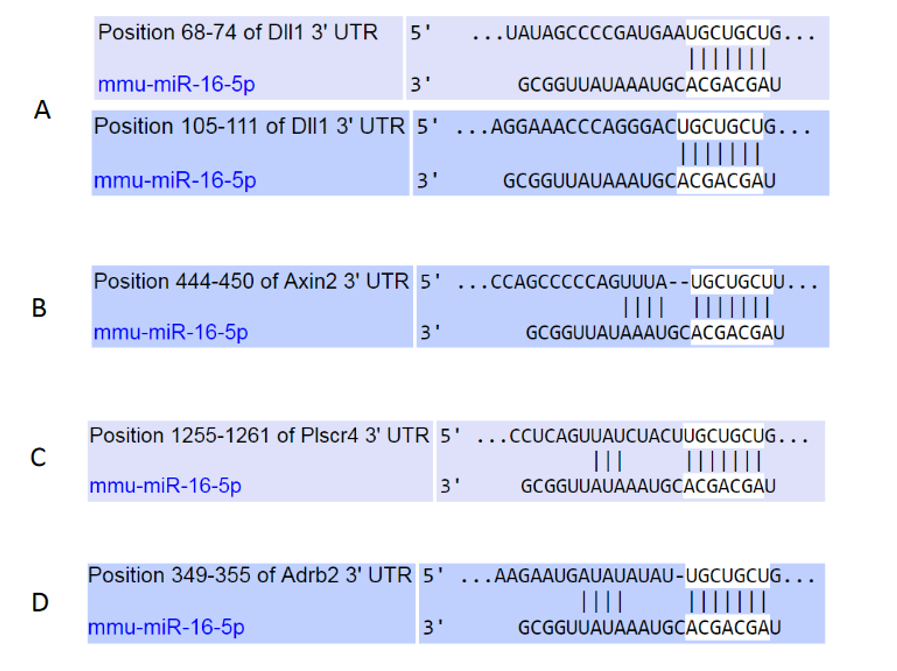


**Figure S9.** Confirmation of the miR-16 complementarity of the miR-16 selected targets—including **A.** DLL1, **B.** Axin2, **C.** PLSCR4, and **D.** ADBR2. Note DLL1 has two miR-16 binding sites.


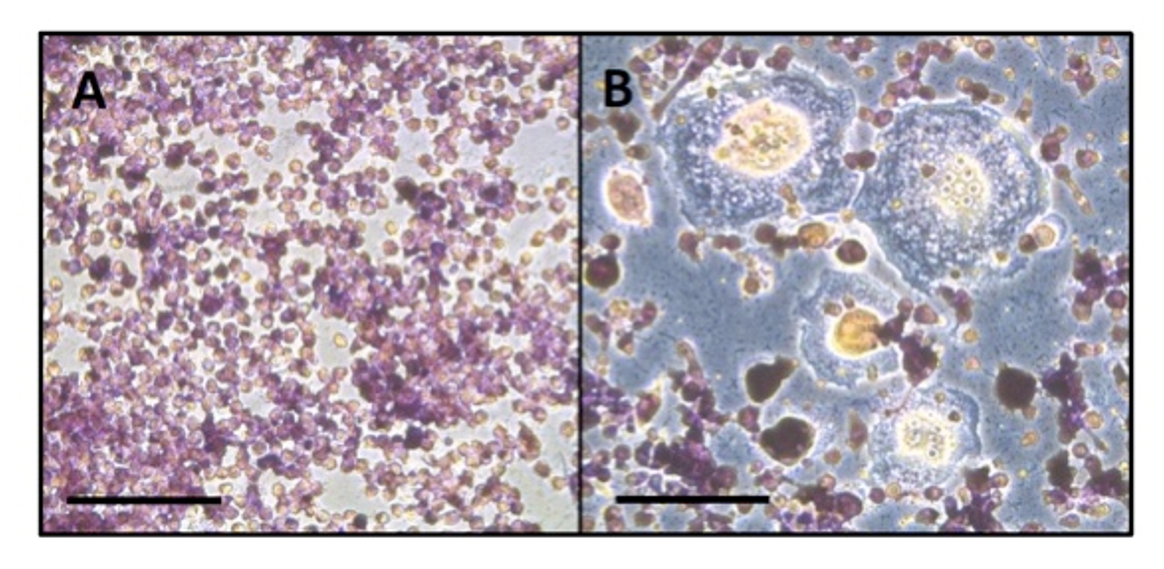


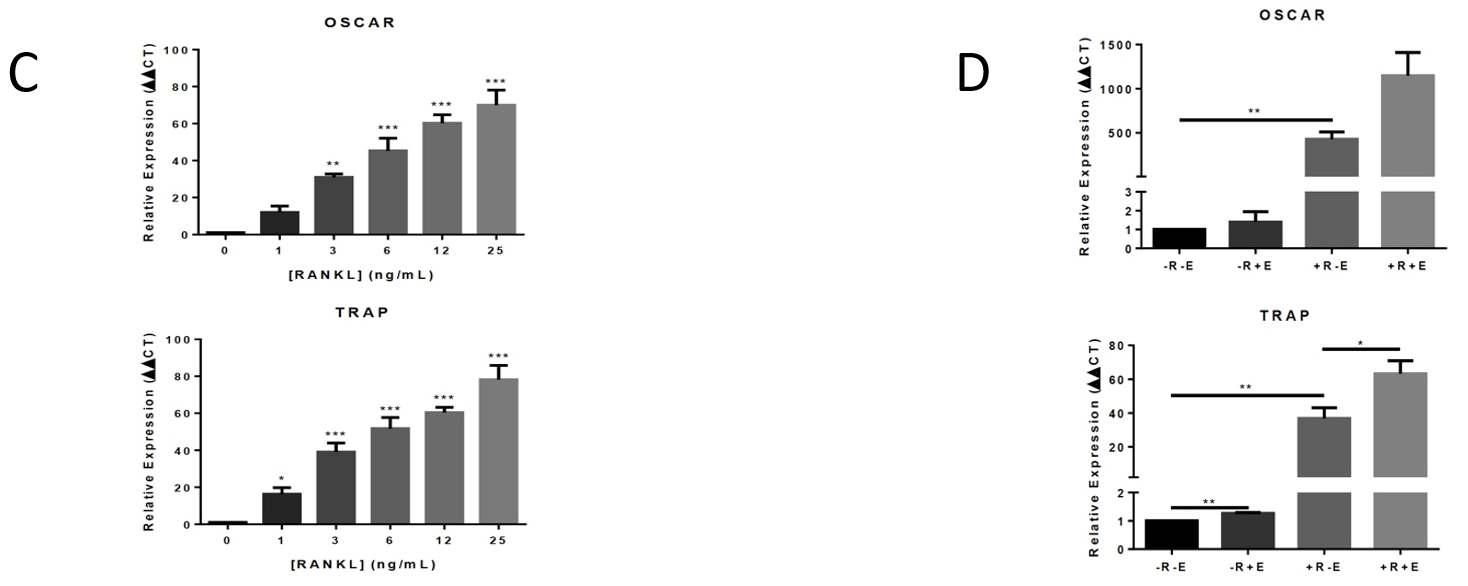


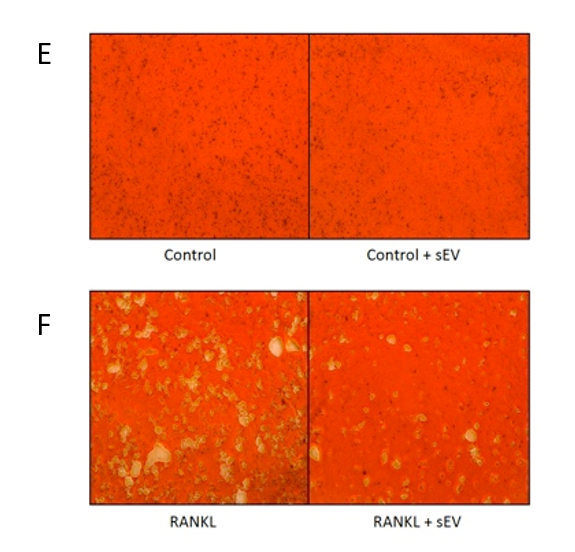


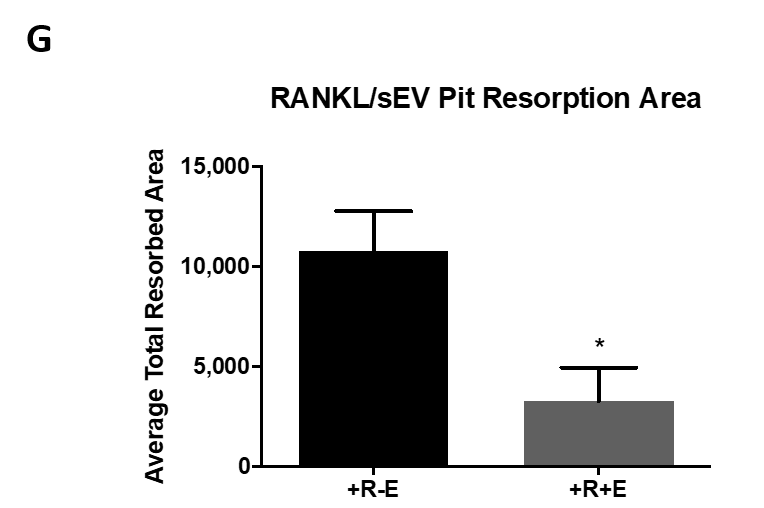


**Figure S10.** EVs Stimulate Osteoclast Differentiation but Reduce Osteoclast Activity. Morphology of RAW Cells. **A.** Microscopy images of TRAP stained RAW cells grown on 6 well plates for 8 days in either DMEM or **B.** DMEM + 25ng/mL RANKL; Scale Bar: 250μm. **C.** Q-PCR expression analysis of osteoclast differentiation genes OSCAR and TRAP in RAW cells treated with RANKL (0–25ng/mL). Graphs show average fold change compared to control +SEM, *n* = 3. Statistical significance assessed by one-way ANOVA, **p* < 0.05, ***p* < 0.01, ****p* < 0.001. **D.** Q-PCR expression analysis of osteoclast differentiation genes OSCAR and TRAP in RAW cells treated with RANKL/sEVs. (25ng/mL) (R) and 200μg/mL of sEVs. Graphs show average fold change compared to control +SEM, *n* = 3. Statistical significance assessed by unpaired test, **p* < 0.05, ***p* < 0.01. **E–G.** Pit formation assay on RANKL and RAW cells treated treated with 25ng/ml of RANKL and 200ng/ml of sEVs. **E.** Control and sEV treated cells in the absence of RANKL. **F.** Control and sEV treated cells with RANKL. **G.** Average resorbed area of control and sEV treated cells (-E / +E) with RANKL (R). Graphs show average +SEM, *n* = 3. Statistical significance assessed by unpaired ttest. * *p* < 0.05.


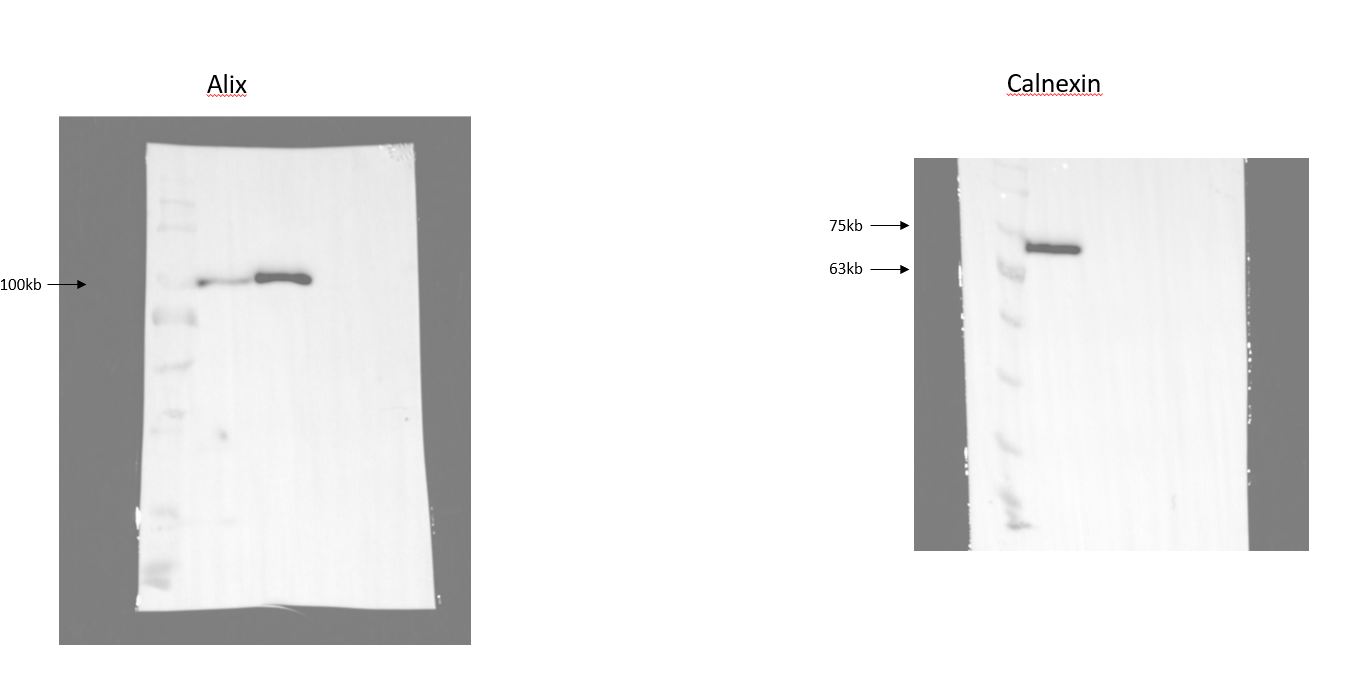


**Figure S11.** Characterisation of PC3 sEVprotein. Western blot of exosome characterisation proteins; ALIX and Calnexin from PC3 whole cell lysate (CL) (20μg) and PC3 sEV lysate (EXO) (20μg). Densitometry analysis is given underneath.

**Table 1.** Heat map gene list—provided separately, attached as an Excel File.

**Text S1.** Supplemental materials.

**Polymerase Chain Reaction**

All qPCR reactions were prepared in MicroAmpFast Optical, barcoded,96 well plates (Thermo Fisher Scientific, Massachusetts, USA) using clear optical adhesive seals (PrimerDesign, Southampton, UK).

SYBR Green qPCR

SYBR^®^ Green qPCR was performed on mRNA generated from the mRNA reverse transcription GoScriptTM Reverse Transcription Mix, Oligo(dT) (Promega). For each reaction, 2μL of diluted cDNA was combined with 8μL of mastermix containing the following components:


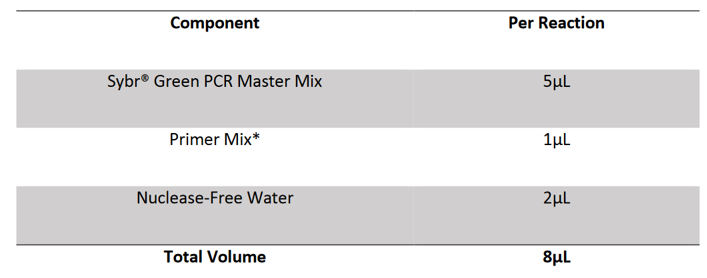


Primer Mix was a pre-made mix of 1μL forward primer, 1μL reverse primer and 38μL nuclease free water. Plates were placed in a StepOne Plus Real-Time PCR System Cycler (Thermo Fisher Scientific, MA, USA) under the following cycling conditions:


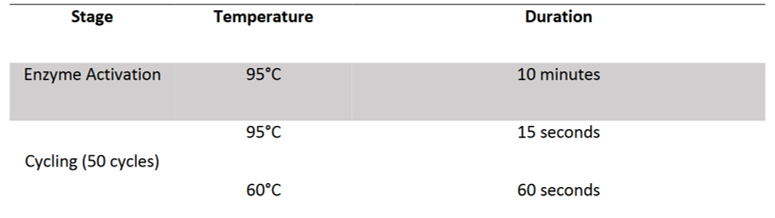


CT values were recorded and gene expression was then quantified using the ΔΔCT method normalised to GAPDH and RPL-19 housekeeping genes for human cells and HPRT and β-Actin for mouse cells.

Primers (designed via the NCBI Primer design website, oligos from EuroFins, UK).

Primers used:


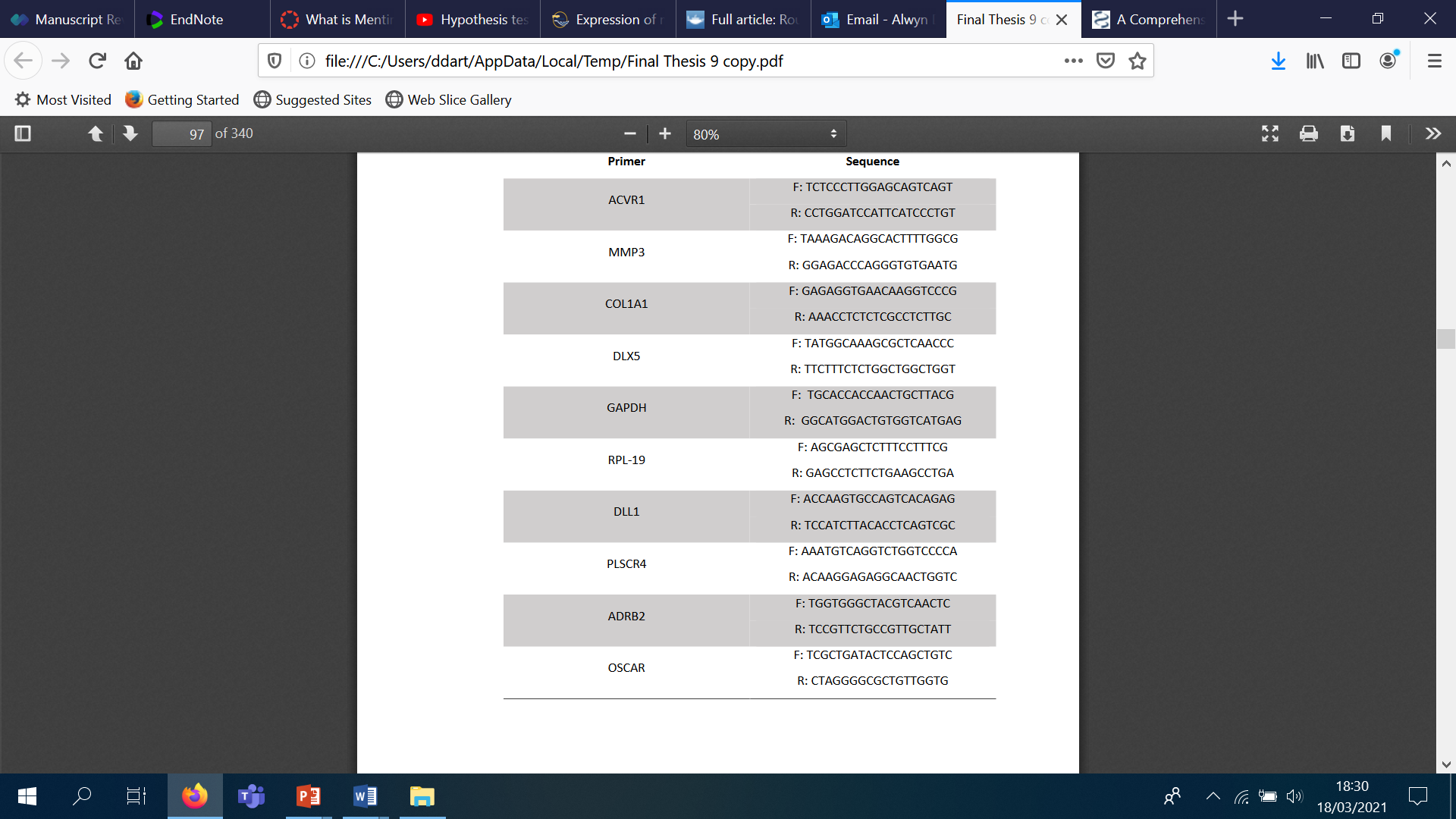


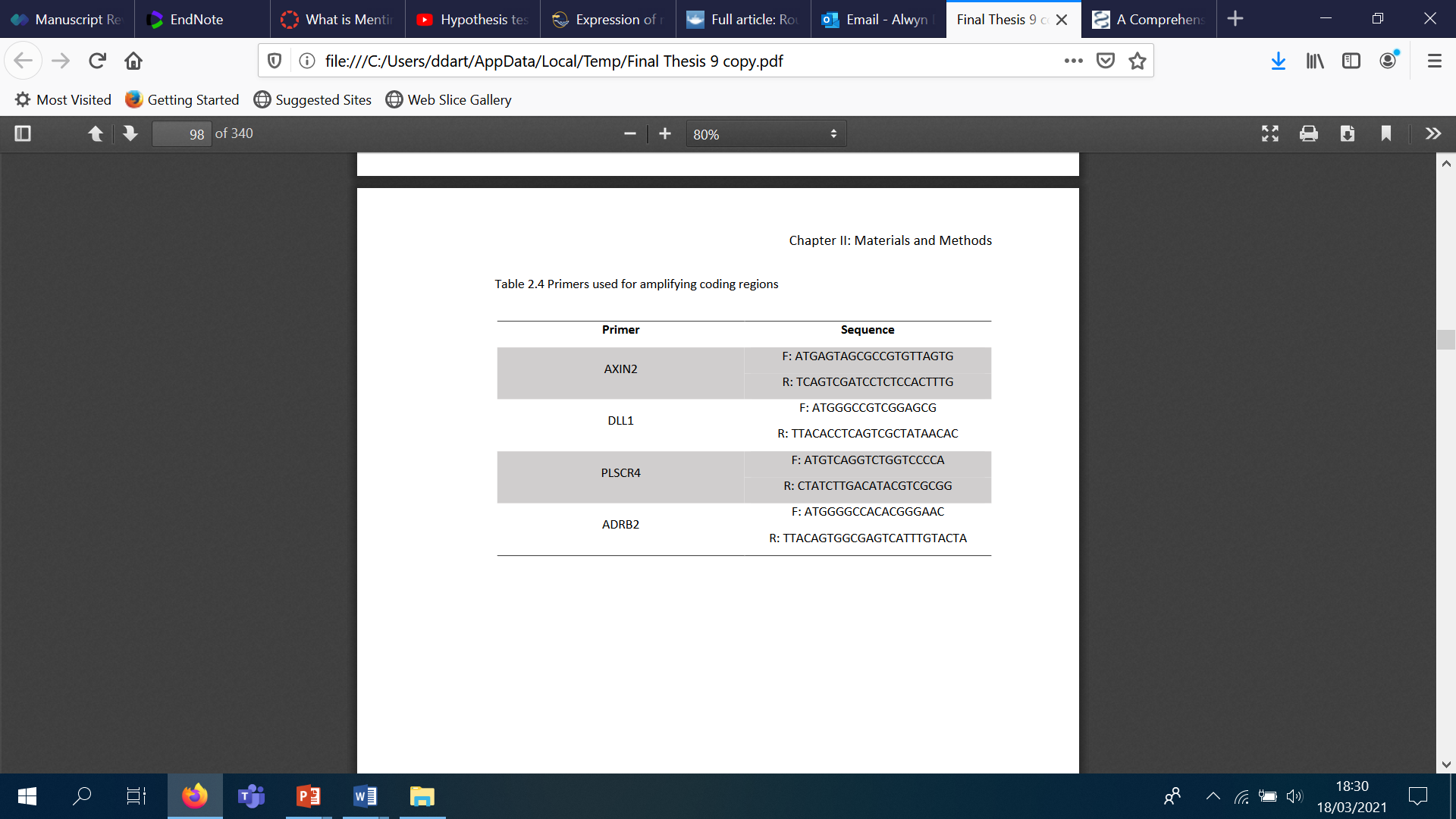


Taqman qPCR Assays (Thermo Fisher)

Method:

Taqman^®^ qPCR was also performed on mRNA generated from the mRNA reverse transcription. For each reaction, 2μL of cDNA (diluted 1:5) was combined with 8μL of mastermix containing the following components:


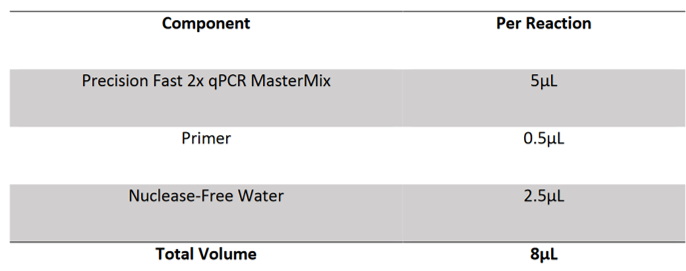


Plates were placed in a StepOnePlus Real-Time PCR System Cycler (Thermo Fisher Scientific, Massachusetts, USA) under the following cycling conditions:


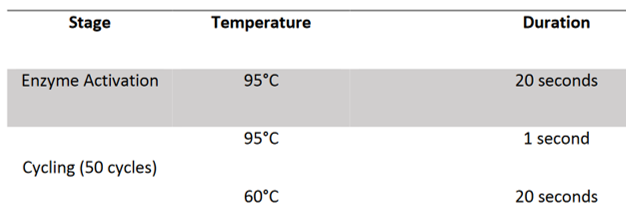


Taqman probes used:


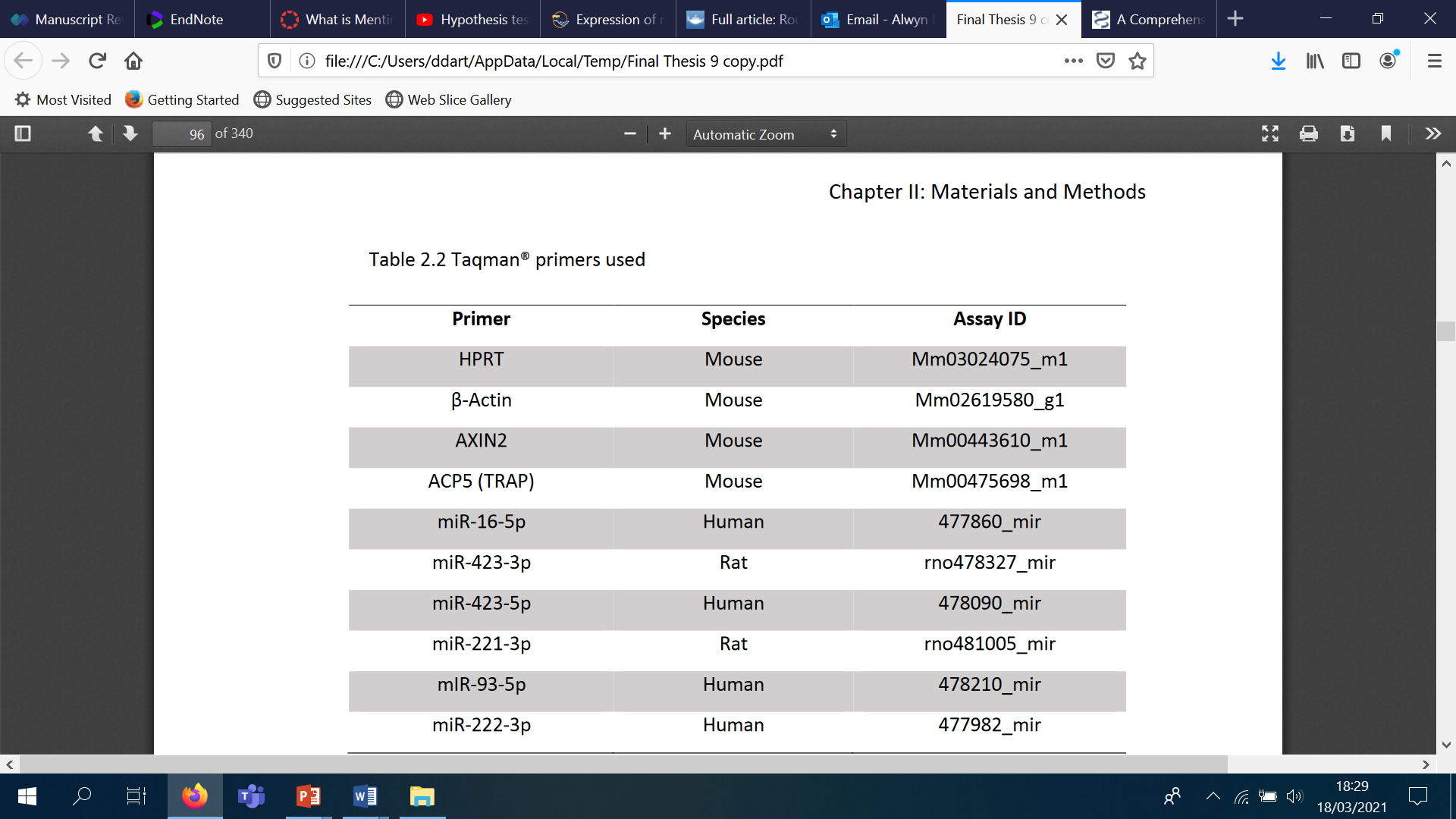


Western Blotting

Antibodies used


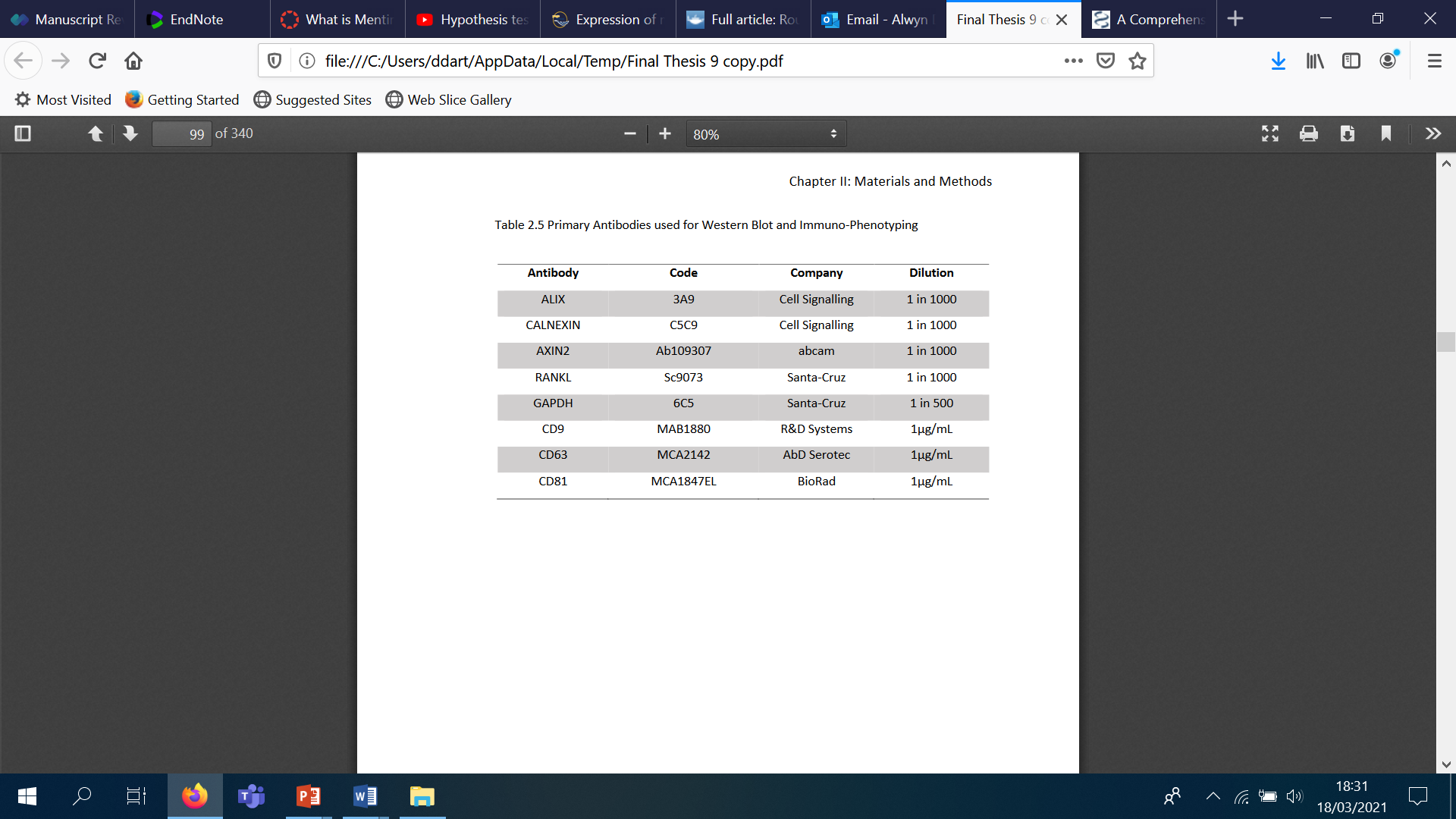


Method:

Membranes were blocked using 5% (w/v) powdered milk (Marvel) in TBS-T for 1 hour. Membranes were incubated with primary antibody overnight in TBS-Twith 3% (w/v) powdered milk solution and 0.1% (v/v) Tween-20, under rotation at 4°C (for primary antibody dilutions see table above). The following day, membranes were washed 3 times with 0.1% (v/v) TBS-T, under rotation at RT for 10 minutes. Membranes were then incubated with HRP-labelled secondary antibodies (Sigma), diluted 1:1000 in TBS-T with 3% (w/v) powdered milk solution, under rotation at RT for 1 hour. Membranes were then washed 2 times with 0.1% (v/v) TBS-Tween-20 and a final time with TBS, under rotation at RT for 10 minutes. Membranes were developed for 3 minutes in EZ-ECL solution (Biological Industries) prepared by mixing equal parts solution A and B. Images were captured using the G-BOX (Syngene). Semi-quantitative analysis was performed using Image J software (National Institute of Health, NY, USA).

Supplemental Methods

sEV Isolation

PC3 cells were cultured in Bioreactor Flasks, CELLine AD 1000 (Integra). Bioreactor flasks are dual chamber flasks, allowing large quantities of cells to grow in a compartment separated by a 10kDa membrane. This compartment holds a maximum of 20mL of media, allowing for extremely concentrated media to be collected. Above the cell compartment, a media compartment holds 0.5–1L of medium. These two chambers ensure delivery of fresh nutrients to the cells through the semi-permeable membrane, whilst allowing a build-up of larger molecules within the cell compartment, in this case, sEVs.

Bioreactor Flask Setup

1% (v/v) antibiotics (penicillin-streptomycin 10,000U/mL) was added to a fresh bottle of medium. 15mL was removed and 5% (v/v) FBS was added to the remaining medium. 50mL of this medium was added to the inside of the bioreactor flask for 5 minutes to wet the semi-permeable membrane and allow it to equilibrate. At least 25x10^6^cells were collected from T75 flasks and resuspended in the 15mL of serum free medium decanted prior. 5% (v/v) exosome depleted FBS (Thermo Fisher Scientific, Massachusetts, USA) was added to the cell suspension and all 15mL was added to the cell compartment using a serological pipette. The remaining media (roughly 500mL) was then added to the media compartment and cells were left for 10 days.

Media Collection

Cell compartment media was collected weekly. Outer chamber media was also changed weekly in coordination with collection chamber media. For collection of inner chamber and changing of outer chamber media, a fresh bottle of DMEM/F-12 was pre-warmed to room temperature. All media from the collection chamber was removed using a 20mL serological pipette and added to a sterile 20mL universal container. 1% (v/v) antibiotics (penicillin-streptomycin 10,000U/mL) was added to the bottle of medium and 45mL was decanted into a sterile 50mL falcon tube. Ten millilitres of this was then used to wash the inner chamber 3 separate times using a fresh 20mL serological pipette, leaving 15mL. 5% (v/v) of exosome depleted FBS (Thermo Fisher Scientific, Massachusetts, USA) was added to the remaining 15mL of media. A final fresh 20mL serological pipette was used to add this 15mL of media into the collection chamber. The outer chamber was then emptied and discarded. 5% (v/v) of regular FBS was added to the remaining 455mL of media.

This media was then carefully poured into the outer chamber of the bioreactor flask, and both lids were tightened. The universal container, containing the collected media was span twice at 2000xg for 5 minutes, and finally at 4000 × g for 15 minutes to remove debris, pouring into fresh universal containers between each centrifugation. The media was then filtered through a 0.8μm filter and subsequently a 0.22μm filter using a 20mL syringe under sterile conditions and stored in a universal container at −80°C. For the sEV isolation, media was defrosted in a 37°C water bath and the ultracentrifuge (Beckmann Coulter, Optima LE-80K) was cooled to 4°C. Media was then transferred to QuickSeal 33mL centrifuge tubes (Beckmann Coulter). A 30% sucrose/D2O cushion consisting of 7.5g sucrose and 22.5g D2O was carefully added to the bottom of the QuickSeal 33mL centrifuge tubes using a spinal needle pouring under gravity, underneath the media, being careful not to disturb the separate layers. Once all air was removed from the tube, the tips of the tube were sealed using a soldering iron and casting dye to create a uniform, air-tight seal. Media then underwent ultracentrifugation at 100,000 × g for 60 minutes on a Beckmann Coulter SW32 swing arm rotor at 4°C. The sucrose cushion was then removed from the ultracentrifuge tubes using a syringe and spinal needle and diluted in enough PBS to fill another ultracentrifuge tube. The sucrose cushion/PBS mix was then added to a fresh ultracentrifuge tube using a spinal needle under gravity. Media was then ultracentrifuged again at 100,000 × g for a further 60 minutes at 4°Con a 70Ti fixed arm rotor to pellet the sEVs. The sEV pellet was then resuspended in 500μL of PBS.

sEV Validation

Nano Particle Analysis

Nanoparticle Tracking Analysis (NTA) is a technique used to measure nanometre sized particles of 10–2000nm in liquids, under flow. NTA uses a high powered 488nm LM14 laser module which is scattered by nanoparticles flowing through the sample chamber in suspension. This scattering is recorded by a highly sensitive digital camera (OrcaFlash 2.8, Hamamatsu C11440, Hamamatsu City, Japan). NTA (Malvern Instruments, Malvern, UK) utilises the scattering of this light to determine the size of the nanoparticle over multiple frames as well as Brownian motion; the random motion of nanoparticles in suspension dependant on the temperature and viscosity of the liquid. The NanoSight TMNS300 machine was calibrated using 100nm latex nanobeads (Malvern Instruments, Malvern, UK). For NTA, particles were suspended in particle free water (Fresenius Kabi, Runcorn, UK) to a concentration of 2 × 10^8^and 9 × 10^8^particles/mL. Samples were administered under a constant speed using a NanoSight syringe pump set to 50, and temperatures were kept at a constant 25°C. Tracking videos of 30 seconds were used and each sample had a total of 6 replicates. Videos were analysed using NTA software (version 3.1), with the camera sensitivity and detection threshold set to 14–16 and 1–3 respectively.

Protein Concentration Assay

The protein concentration of sEV samples was assessed using a Bio-Rad DCTM Protein Assay kit (Bio-Rad Laboratories, Hemel-Hempstead, UK). Samples were diluted 1:8 in PBS and compared to a standard curve from 0–2000μg/mL of BSA.

Immuno-Phenotyping Assay

SEVs were diluted in PBS and 1μg was added to each well of a High Protein Binding ELISA Strip 96 well plate (Greiner Bio-One, Frickenhausen, Germany). SEVs were incubated in the wells at 4°C overnight. Wells were washed 3 times using TRIS-wash buffer (Kaivogen, Finland). 1% BSA (w/v in PBS) blocking solution was added for 2 hours at room temperature and wells were subsequently washed 3 times. Primary antibodies (1μg/mL) were added and incubated for 2 hours at room temperature and subsequently washed 3 times. Biotinylated secondary antibody (Perkin Elmer, Waltham, MA, USA) was diluted to 200ng/mL in 0.1% BSA (w/v in PBS) and incubated in wells for 1 hour at room temperature. Wells were washed again 3 times and a Europium-Streptavidin conjugate (Perkin Elmer) in assay buffer (Kaivogen) was added and incubated at room temperature for 45 minutes. Well were finally washed 6 times and enrichment intensifier (Kaivogen) was added and incubated at room temperature for 5 minutes. Time-resolved fluorescence (TRF) was measured using a PHERAstar FS Microplate Reader (BMG Labtech, Bristol, UK).

Size Exclusion Chromatography

A 30cm column (BioRad) was filled with Sepharose CL-2B (Sigma) and washed thoroughly with PBS. 1ml of DMEM or conditioned media was loaded and allowed to flow through under gravity. 30 × 500 μl fractions were collected. For RNA analysis, fractions were extracted using Trizol-LS (liquid sample) from Sigma.

Oligo Transfection

Mimics of hsa-miR-16a, and a scramble miR control were purchased from Sigma-Aldrich (Dorset, UK). MiRNA oligos were transfected using a LipofectamineTM3000 kit (Thermo Fisher Scientific, MA, USA) following the manufacturers protocol for RNA.

MiR-reporter constructs

The following oligos were synthesised from Eurofin UK. The reporters were constructed according to the pMir-Glo vector from Promega.


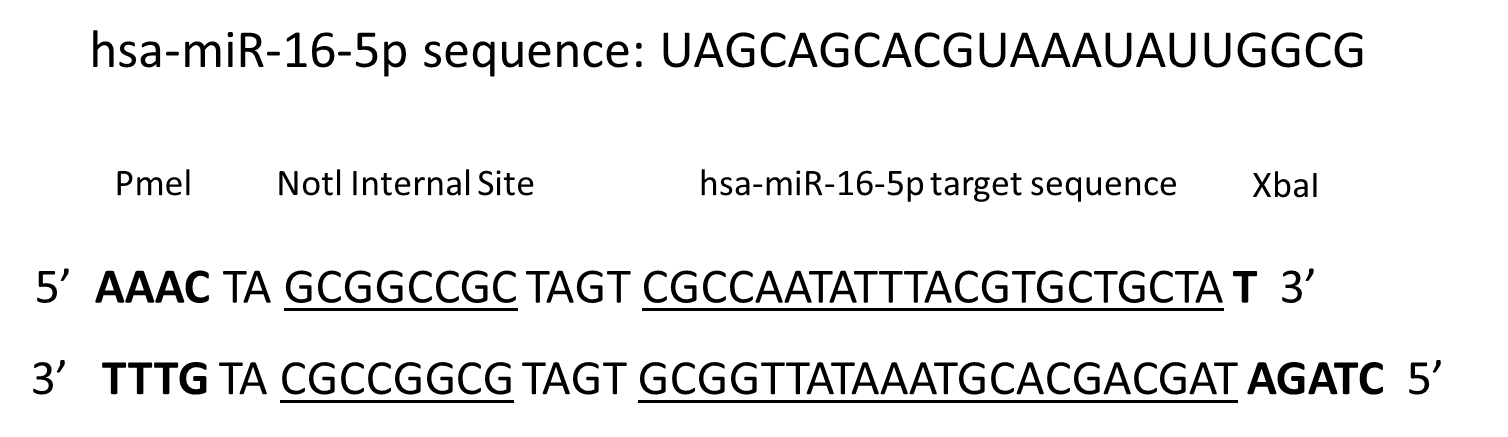


References

1. Muguruma, Y.; Hozumi, K.; Warita, H.; Yahata, T.; Uno, T.; Ito, M.; Ando, K. Maintenance of Bone Homeostasis by DLL1-Mediated Notch Signaling. *J. Cell. Physiol.* **2016**, *232*, 2569–2580, doi:10.1002/jcp.25647.
2. Zanotti, S.; Smerdel-Ramoya, A.; Stadmeyer, L.; Durant, D.; Radtke, F.; Canalis, E. Notch Inhibits Osteoblast Differentiation and Causes Osteopenia. *Endocrinol.* **2008**, *149*, 3890–3899, doi:10.1210/en.2008-0140.
3. Yan, Y.; Tang, D.; Chen, M.; Huang, J.; Xie, R.; Jonason, J.H.; Tan, X.; Hou, W.; Reynolds, D.; Hsu, W.; et al. Axin2 controls bone remodeling through the -catenin-BMP signaling pathway in adult mice. *J. Cell Sci.* **2009**, *122*, 3566–3578, doi:10.1242/jcs.051904.
4. McGee-Lawrence, M.E.; Li, X.; Bledsoe, K.L.; Wu, H.; Hawse, J.R.; Subramaniam, M.; Razidlo, D.F.; Stensgard, B.A.; Stein, G.S.; van Wijnen, A.J.; et al. Runx2 Protein Represses Axin2 Expression in Osteoblasts and Is Required for Craniosynostosis in Axin2-deficient Mice*. *J. Biol. Chem.* **2013**, *288*, 5291–5302, doi:10.1074/jbc.m112.414995.
5. Andersen, R.K.; Zaher, W.; Larsen, K.H.; Ditzel, N.; Drews, K.; Wruck, W.; Adjaye, J.; Abdallah, B.M.; Kassem, M. Association between in vivo bone formation and ex vivo migratory capacity of human bone marrow stromal cells. *Stem Cell Res. Ther.* **2015**, *6*, 1–14, doi:10.1186/s13287-015-0188-9.
6. Kubista, B.; Klinglmueller, F.; Bilban, M.; Pfeiffer, M.; Lass, R.; Giurea, A.; Funovics, P.T.; Toma, C.; Dominkus, M.; Kotz, R.; et al. Microarray analysis identifies distinct gene expression profiles associated with histological subtype in human osteosarcoma. *Int. Orthop.* **2010**, *35*, 401–411, doi:10.1007/s00264-010-0996-6.
7. Ma, Y.; Nyman, J.S.; Tao, H.; Moss, H.H.; Yang, X.; Elefteriou, F. β2-Adrenergic Receptor Signaling in Osteoblasts Contributes to the Catabolic Effect of Glucocorticoids on Bone. *Endocrinol.* **2011**, *152*, 1412–1422, doi:10.1210/en.2010-0881.
